# EGFR/Ras Signaling Controls *Drosophila* Intestinal Stem Cell Proliferation via Capicua-Regulated Genes

**DOI:** 10.1371/journal.pgen.1005634

**Published:** 2015-12-18

**Authors:** Yinhua Jin, Nati Ha, Marta Forés, Jinyi Xiang, Christine Gläßer, Julieta Maldera, Gerardo Jiménez, Bruce A. Edgar

**Affiliations:** 1 Deutsches Krebsforschungszentrum (DKFZ) - Zentrum für Molekulare Biologie der Universität Heidelberg (ZMBH) Allianz, Heidelberg, Germany; 2 Biochemie-Zentrum der Universität Heidelberg (BZH), Heidelberg, Germany; 3 Institut de Biologia Molecular de Barcelona-CSIC, Parc Científic de Barcelona, Barcelona, Spain; 4 Institució Catalana de Recerca i Estudis Avançats (ICREA), Barcelona, Spain; Fred Hutchinson Cancer Research Center, UNITED STATES

## Abstract

Epithelial renewal in the *Drosophila* intestine is orchestrated by Intestinal Stem Cells (ISCs). Following damage or stress the intestinal epithelium produces ligands that activate the epidermal growth factor receptor (EGFR) in ISCs. This promotes their growth and division and, thereby, epithelial regeneration. Here we demonstrate that the HMG-box transcriptional repressor, Capicua (Cic), mediates these functions of EGFR signaling. Depleting Cic in ISCs activated them for division, whereas overexpressed Cic inhibited ISC proliferation and midgut regeneration. Epistasis tests showed that Cic acted as an essential downstream effector of EGFR/Ras signaling, and immunofluorescence showed that Cic’s nuclear localization was regulated by EGFR signaling. ISC-specific mRNA expression profiling and DNA binding mapping using DamID indicated that Cic represses cell proliferation via direct targets including *string (Cdc25)*, *Cyclin E*, and the ETS domain transcription factors *Ets21C* and *Pointed (pnt)*. *pnt* was required for ISC over-proliferation following Cic depletion, and ectopic *pnt* restored ISC proliferation even in the presence of overexpressed dominant-active Cic. These studies identify Cic, Pnt, and Ets21C as critical downstream effectors of EGFR signaling in *Drosophila* ISCs.

## Author Summary

Studies suggest that epidermal growth factor receptor (EGFR) signaling activation is a causal driver of many stem cell-derived epithelial cancers, including colorectal cancer. As in the human intestine, epithelial renewal in *Drosophila* intestine is orchestrated by intestinal stem cells (ISCs). EGFR signaling also plays an important role in regulating ISC proliferation in flies. However, the mechanism by which EGFR/Ras/MAPK signaling promotes ISC proliferation is poorly understood. Here we demonstrate that the transcriptional repressor, Capicua (Cic), mediates these functions of EGFR signaling. We found that the critical role of Cic as a negative regulator of cell proliferation in the fly midgut is consistent with its tumor suppressor function in mammalian cancer development. The direct target genes of Cic were identified by ISC-specific mRNA expression profiling and DNA binding mapping (DamID) method. Cic represses cell proliferation via regulating *string (stg)*, *Cyclin E (CycE)*, and the ETS domain transcription factors *Ets21C* and *pointed* (*pnt*). Using genetic tests we show that these interactions are meaningful for regulating stem cell proliferation. Combining our knowledge of Cic with what was previously known about CIC in tumor development, we propose that human CIC may regulate Ets transcription factors and cell cycle genes in Ras/MAKP-activated tumors.

## Introduction

EGFR/Ras/MAPK signaling has diverse functions in regulating cell proliferation, growth, differentiation and survival in most animal cells [1]. Abundant studies also indicate that epidermal growth factor receptor (EGFR) activation is a causal driver of many cancers, including breast, lung, brain, and colorectal cancer [2]. Similarly, activating mutations in KRAS and BRAF, which are essential downstream effectors of the EGFR, are among the most common mutations found in a very wide range of human cancers [3,4]. However, despite much study, many questions remain to be answered to fully understand the impact of EGFR and its downstream effectors during normal cell function and in carcinogenesis. As many epithelial cancers arise through dysregulation of the stem cell self-renewal and homeostatic maintenance of the epithelium [5], understanding the precise functions of EGFR signaling in epithelial homeostasis is very important.

The *Drosophila* midgut is an outstanding model system to study the basis of epithelial homeostasis due to its simple structure, similarity to the mammalian intestine, and powerful genetics. As in the mammalian intestine, epithelial turnover in the fly midgut is carried out through a dynamic process mediated by intestinal stem cells (ISCs). ISCs undergo cell division to renew themselves and give rise to transient cells called enteroblasts (EBs), which can further differentiate into either absorptive enterocytes (ECs) or secretory enteroendocrine (EE) cells. When damaged or aged cells are lost from the fly’s gut epithelium, ISCs respond by dividing to replenish the epithelium [6,7,8]. During this response multiple *Drosophila* EGFR ligands, namely *spitz (spi)*, *vein (vn)*, and *keren (krn)* are induced in progenitor cells (EBs and ISCs), visceral muscle (VM) and ECs respectively. Thereby, the EGFR signaling pathway is activated in ISCs. This promotes ISC growth, division and midgut epithelial regeneration [9,10,11]. ISCs defective in EGFR signaling cannot grow or divide, are poorly maintained, and are unable to support midgut epithelial replenishment after enteric infection by the bacteria *Pseudomonas entomophila (P*.*e*.*)* [11] or *Erwinia carotovora carotovora 15 (ECC15)* [12]. Interestingly, the critical role of EGFR signaling in the *Drosophila* intestine is consistent with its role during mammalian gut homeostasis and colorectal cancer development [10,11,12,13]. EGFR signaling is required for murine ISC growth [14,15], and the deletion of Lrig1, a negative feedback regulator of EGFR signaling, causes excessive ISC proliferation [16]. Furthermore, adenoma formation in *Apc* ^*min/+*^ mice was severely impaired in a genetic background with partial loss of function of EGFR (*Egfr* ^*wa2*^) [17].

Despites its importance, the mechanism by which EGFR/Ras/MAPK signaling promotes ISC proliferation is poorly understood in this cell type. Indeed, despite decades of intensive study, the precise linkage between EGFR/Ras/MAPK signaling and cell growth and division is surprisingly obscure for animal cells in general [3]. Textbook models highlight a prevailing model in which EGFR/Ras signaling controls cell proliferation via a Ras-Myc-CyclinD-Rb pathway [18,19]. While this may have relevance in some human cancers it is clearly not the case in normal *Drosophila* cells, and so other mechanisms should be sought and characterized.

One potentially important downstream effector of EGFR signaling is the HMG-box transcriptional repressor Capicua (Cic). This highly conserved DNA binding factor has been shown to act downstream of receptor tyrosine kinase (RTK)/Ras/MAPK signaling in *Drosophila* eye and wing imaginal discs, embryos, and ovaries [20,21,22,23] where it regulates diverse RTK-dependent processes including cell proliferation, specification, and pattern formation. Cic orthologs from invertebrate and vertebrate species share two well-conserved regions: the HMG-box, presumed to mediate DNA binding at target promoters [21] and a C-terminal domain [24]. The C-terminal region of *Drosophila* Cic contains a “C1” motif important for repressor activity, and a “C2” motif that functions as a MAPK docking site responsible for downregulation of Cic following the activation of RTK signaling [25]. It has been proposed that MAPK phosphorylates Cic in its C2 motif, and that phosphorylated Cic is either degraded or re-localized to the cytoplasm [25]. Cic downregulation controlled by Torso and EGFR signaling varies in different *Drosophila* tissues [24]. For example, Torso RTK signaling, which also works via the Ras/Raf/MAPK pathway, apparently increases the rate of Capicua degradation by promoting its accumulation in the cytoplasm [26]. EGFR signaling has been reported to regulate Cic protein in distinct ways in different tissues. Wing and eye discs cell clones mutant for *Egfr* or *Ras* showed elevated levels of Cic protein [20,27]. In the ovary, in contrast, Cic protein localized to the cytoplasm in cells in which EGFR signaling was active, but in nuclei in cells in which EGFR signaling was inactive [25]. A recent study suggested that Cic actually undergoes a two-step process in releasing its target gene repression: slower changes in nuclear localization occur after a faster reduction of Cic repressor activity [28]. In cultured human cells, EGF stimulated dissociation of human CIC from importin-α4 (also known as KPNA3), an adaptor required for the nuclear import of many proteins. But full length GFP-CIC was nuclear even after EGF stimulation, and the N-terminal half of the CIC protein was found to be nuclear, even though it does not bind to importin-α4. Hence the biological significance of the CIC:importin association remains unclear [29].

CIC, the human homolog of *Drosophila Cic*, has been implicated in several human diseases including spinocerebellar ataxia type 1 (SCA1) neuropathology, oligodendroglioma (OD) [30] and Ewing-like sarcoma [31]. Human *CIC* is frequently mutated in samples from cancer genome studies such as The Cancer Genomic Atlas (TCGA) (S1 Fig) [32,33]. For instance CIC mutation was reported in 6 out of 7 brain tumors [30], 3 out of 11 breast cancers [34] and 6 out of 72 colorectal cancers [35]. The *Drosophila* work suggests that in these cases CIC loss might have the same downstream consequences (*e*.*g*. cell transformation) as oncogenic activation of the EGFR, RAS or BRAF, but this has not been rigorously evaluated.

During RNAi screening we discovered that depletion of Cic in *Drosophila’s* intestinal stem cells (ISCs) activates these cells for rampant proliferation [11]. Based on previous studies in other fly organs we hypothesized that Cic might act as an obligate repressor downstream of EGFR signaling, itself a central driver of normal ISC proliferation in both flies and mice, as well as in many human colorectal cancers, which are frequently mutant for RAS, BRAF, or CIC. However, until now this hypothesis had not been tested and the underlying mechanisms via which Cic might control ISC proliferation remained undefined. In this report we demonstrate that Cic acts as a critical negative downstream regulator of EGFR signaling to control ISC proliferation. We show that EGFR/Ras activity controls Cic nuclear localization, and we present RNA-Seq and DamID-Seq datasets that together constitute a genome-wide survey of potential Cic target genes in *Drosophila* ISCs. Our analysis indicated that Cic not only directly regulates cell cycle regulators such as *string (cdc25)* and *Cyclin E*, but also the ETS transcription factors *pnt* and *Ets21C*, all of which must be de-repressed to activate ISCs for growth and division.

## Results

### Cic inactivation promotes ISC proliferation

**Fig 1:**
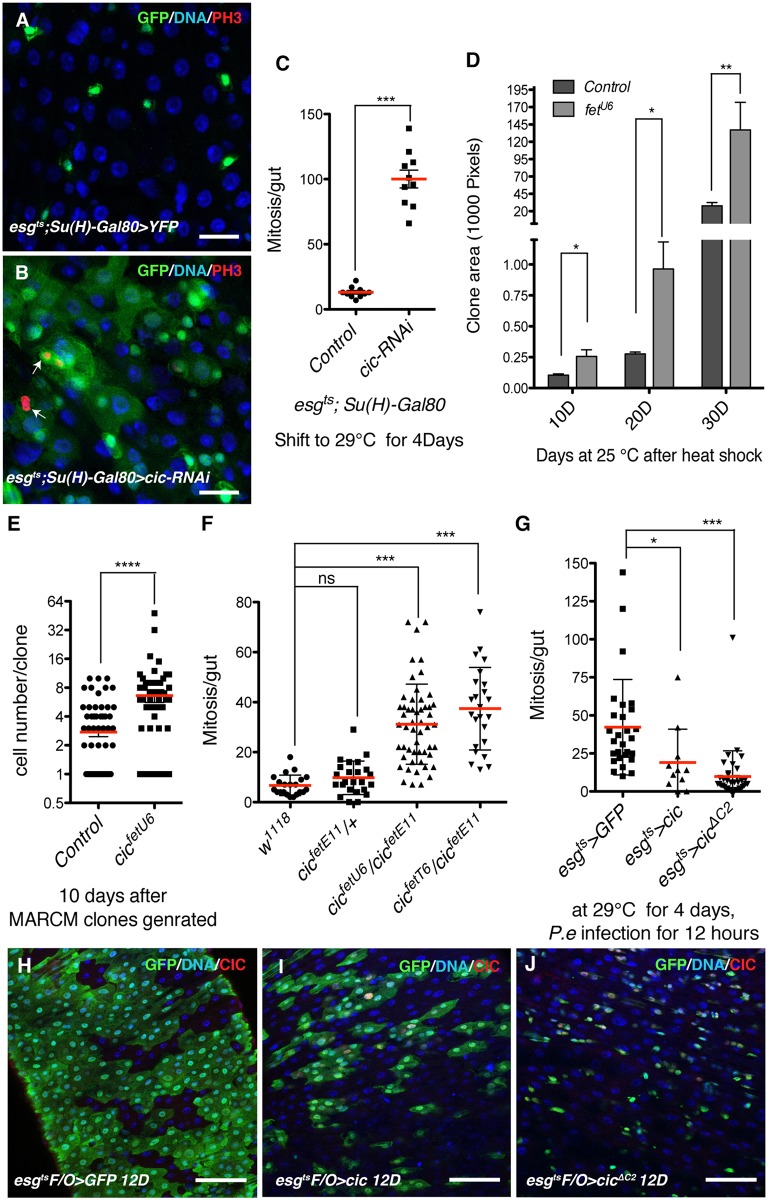
*cic* inactivation promotes ISC proliferation and hyper-activation inhibits ISC proliferation. (A,B) Knock down of Cic in ISCs using the *esg* ^*ts*^ *; Su(H)-Gal80* system. ISCs were marked by YFP (green). Samples were stained with anti-PH3 (red) for mitosis and DAPI (blue) for DNA. (A) Control adult midgut (B) Cic knock down midgut after 4 days induction 29°C. Increases in the number of YFP+ cells are observed in *cic* depleted midguts as was a large increase in mitotic cells. (C) Midguts were scored for PH3+ cells after 4 days of induction of *cic-RNAi*. A strong increase in numbers of ISC mitosis was observed in *cic* knockdown midguts. (D) Clone areas of cic mutant and control WT clones 10, 20, and 30 days after clone induction. Mutant ISCs divided faster and generated bigger clones. (E) Increased number of cells per clone was detected in *cic* mutant clones. Data was quantified 10 days after *cic* mutant clones were generated with the MARCM system. (F) Quantification of pH3-positive cells per adult midgut of the indicated genotype. *cic* transheterozygotes contained significantly more mitotic cells than controls. (G) Quantification of ISC proliferation after 12 hours *P*.*e*. infection. A decreased number of PH3+ cells, representing dividing ISCs, was observed in midguts overexpressing either *cic* or *cic* ^*ΔC2*^ after *P*.*e*. infection. (H-J) Clones generated by the *esg* ^*ts*^ *F/O* system are marked with GFP (green), Cic over-expression was confirmed by anti-Cic (red) staining, and nuclei were visualized by DAPI (blue) staining. (E) Control adult midgut 12 days after clone induction (F) midgut overexpressing Cic (G) midgut overexpressing Cic^ΔC2^ 12 days after clone induction. The size of clones marked by GFP was reduced after Cic or Cic^ΔC2^ overexpression. Statistical significance was determined by Student’s t test (*p<0.05, **p<0.01, ***p<0.001, ****p<0.0001). Error bars represent standard deviations. Scale bars represent 20 μm in A-B and 50 μm in E-G.

To investigate a potential role for Cic in regulating ISC proliferation, we used the *esg-Gal4-UAS-2XEYFP; Su(H)GBE-Gal80*, *tub-Gal80* ^*ts*^ system (henceforth referred as *esg* ^*ts*^ *; Su(H)-Gal80*) to express *UAS-cic-RNAi* specifically in ISCs. After 4 days of *cic-RNAi* induction, a dramatic increase in the number of YFP positive cells (Fig 1A and 1B) and a large increase in ISC mitoses were observed (Fig 1C). Most of the PH3+ cells were YFP+ [YFP+, PH3+ cells = 99.37% (n^midguts^ = 10 midguts, n^cells^ = 994), YFP-, PH3+ cells = 0.63% (n = 10, n^cells^ = 7)], indicating that Cic regulates ISC proliferation cell autonomously. When we used another ISC-specific driver *Dl* ^*ts*^ *(tub-Gal80* ^*ts*^ *UAS-GFP; Dl-Gal4)* to knock down *cic* in ISCs specifically, we not only detected the same overporoliferation phenotype (S3A, S3B and S3E Fig) but also found that most of mitotic cells were GFP+ (S3F Fig).

Increased GFP+ cells and mitoses were also noticed when the *esgGal4 UAS-GFP tub-Gal80* ^*ts*^ system (henceforth referred as *esg* ^*ts*^) was used to express *UAS-cic-RNAi* in ISCs and their undifferentiated daughters, the EBs (S2A–S2B and S3 Figs). To further validate the specificity of this RNAi experiment, GFP-marked ISC clones homozygous for the loss-of-function allele *cic* ^*fetU6*^ [22] were generated using the MARCM system [36] (S2C–S2H Fig). The size of marked ISC clones was quantified at intervals after clone induction by measuring GFP-labeled clone areas. *cic* mutant clones were larger than control clones at all time points assayed (Fig 1D). In addition, the numbers of cells per clone were increased in the *cic* mutant clones (Fig 1E). To further confirm Cic’s function in the midgut, we generated viable transheterozygotes using three different loss-of-function alleles of *cic*. *cic* ^*fetE11*^ is a P-element insertion mutant, while both *cic* ^*fetT6*^ and *cic* ^*fetU6*^ are homozygous lethal EMS alleles [22]. In addition to the EGFR-related extra wing vein phenotype reported previously [27], these transheterozygote mutants showed increased mitoses in their midguts (Fig 1I). As the ISCs are the predominant dividing cell type in *Drosophila* midguts, these data further indicate a role for Cic as an obligate repressor of ISC proliferation.

To investigate the respective requirements of Cic in the ISC and EB cell types, the EB-specific driver *Su(H)* ^*ts*^ *[Su(H)-Gal4*,*UAS-CD8-GFP; tub-Gal80* ^*ts*^ *]* was used to knock down *cic* in EBs. Increased mitoses were observed after depleting *cic* in EBs (S3C–S3E Fig). However, in this case only a few GFP^+^ EBs were observed in mitosis, while most of the dividing cells marked by PH3 were GFP-negative (S3F Fig). These GFP-negative mitotic cells are likely ISCs. These data indicated that Cic has both cell autonomous and non-cell autonomous functions in regulating ISC proliferation. In this study we followed up on Cic’s cell autonomous effects on ISC proliferation, and the non-cell autonomous effect was not investigated further.

### Increased Cic activity inhibits ISC proliferation and midgut epithelial regeneration

To determine whether increased Cic function yields a phenotype similar to that of EGFR loss-of-function, we generated transgenic flies harboring *UAS-cic* ^*ΔC2*^ *-HA or UAS-cic-HA*. Cic^ΔC2^ is a Cic derivative carrying a deletion of the MAPK docking site-C2 motif, and has been shown to be a dominant repressor that escapes inactivation by MAPK [25]. Either *cic* or *cic* ^*ΔC2*^ were over-expressed in progenitor cells using *esg* ^*ts*^, and then the flies were fed *Pseudomonas entomophila (P*.*e*.*)* for 12 hours to generate an enteric infection. ISCs from control midguts, which expressed GFP only, showed regeneration-associated proliferation [8]. In contrast both *cic* and *cic* ^*ΔC2*^ overexpressing midguts displayed an inhibition of regeneration after 12 hours *P*.*e*. infection (Fig 1J). To test if *cic* or *cic* ^*ΔC2*^ overexpression could influence turnover of the midgut epithelium we used the *esg* ^*ts*^ *F/O* system *(esg-Gal4; tubGal80* ^*ts*^ *Act>Cd2>Gal4 UAS-flp UAS-GFP)* [11] to mark all the ISC progeny produced during 12 days of *cic* overexpression. Normally, the posterior midgut epithelium renews it self within about 12 days [8]. Therefore, control midgut epithelia were almost completely replaced by large GFP+ clones that formed during 12 days. However, in the gain-of-function Cic conditions, growth of GFP-marked clones was significantly decreased, indicating that gut epithelial renewal was greatly suppressed (Fig 1H–1J).

### Cic regulates ISC proliferation as a downstream effector of EGFR/Ras signaling

EGFR activates ISCs for growth and division via Ras/Raf/MAPK signaling. When an activated form of the EGFR (λTOP) [37] or activated Ras (*Ras* ^*V12G*^) [38] is ectopically expressed in progenitor cells, ISC division is dramatically induced. Conversely, EGFR suppression by inducing *Egfr-RNAi*, *Ras-RNAi*, *or MEK-RNAi* in progenitor cells almost completely inhibits ISC division and growth [11,12]. Furthermore, inhibition of EGFR signaling suppresses the activation of ISC divisions after *P*.*e*. infection [10,11]. As demonstrated above, Cic knockdown and overexpression phenocopy these EGFR overexpression or knockdown phenotypes, respectively, suggesting that Cic may act as a downstream effector in the EGFR signaling in ISCs.

**Fig 2:**
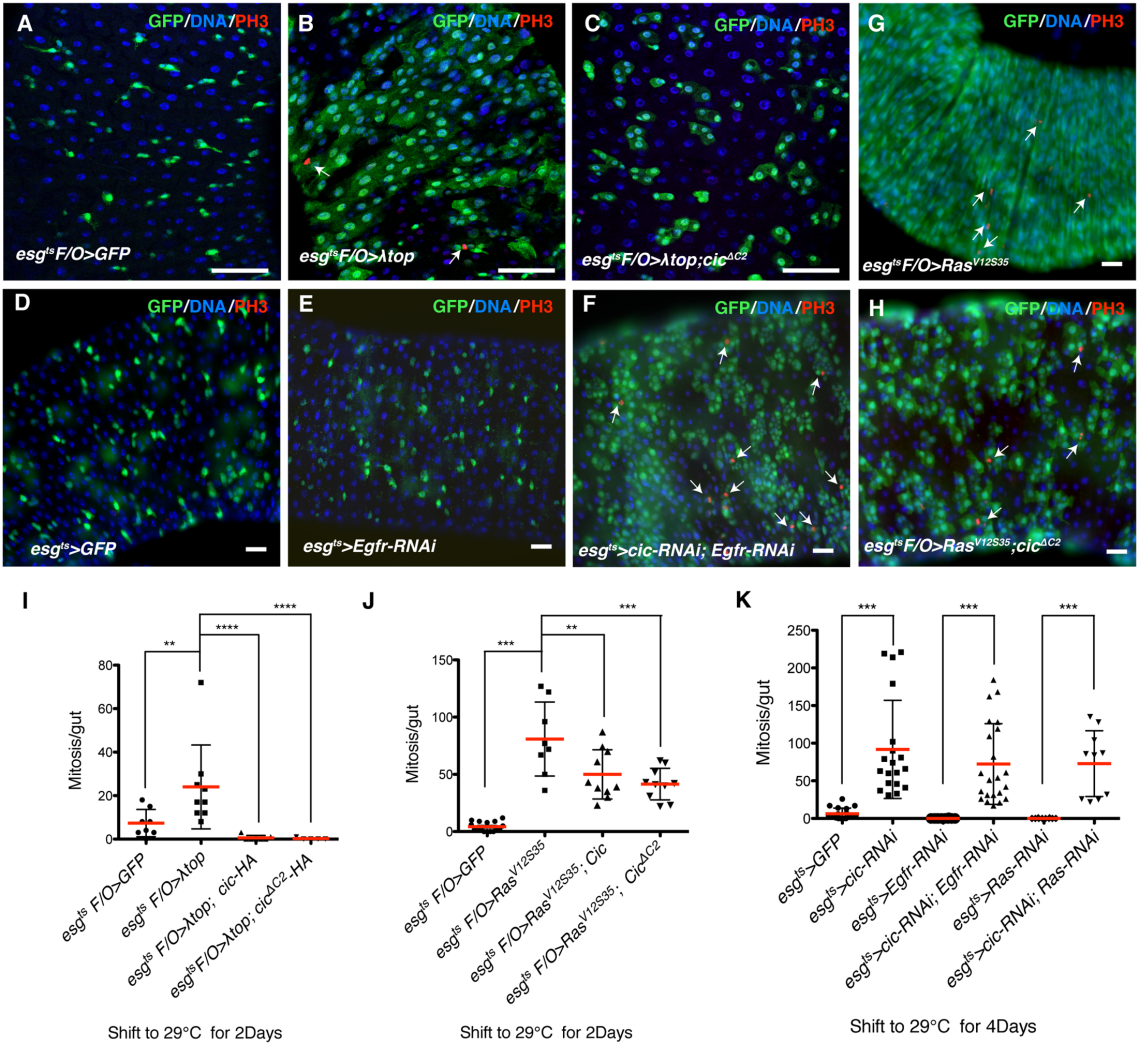
Cic regulates ISC proliferation as a downstream effector of EGFR signaling. (A–C) Results of the *λtop* and *cic* epistasis tests, carried out using the *esg* ^*ts*^ *F/O* system, to co-express the indicated transgenes with GFP for 2 days at 29°C. (A) Control of adult midgut (B) *λtop* overexpresssing midgut (C) *λtop* and *cic* ^*ΔC2*^ co-overexpresssing midgut. GFP+ clones (green) expressing *λtop* were much smaller when *cic* ^*ΔC2*^ was co-overexpressed. Samples stained with anti-PH3 (red) and DAPI (blue). (D-F) Results of the epistasis test between *cic* and *egfr*, carried out using the *esg* ^*ts*^ system to express the indicated transgenes for 4 days at 29°C. (D) Control adult midgut, (E) *Egfr-RNAi* expressing midgut, (F) *Egfr-RNAi* and *cic-RNAi* co-expressing midgut. The number of GFP+ cells (green) still promoted by depleting *cic* in EGFR/Ras inactivated background. Samples were stained with anti-PH3 (red) and DAPI to visualize nuclei. (G-H) Results of epistasis tests between *Ras* ^*V12S35*^ and *cic*, carried out using the *esg* ^*ts*^ *F/O* system. The transgenes were induced for 2 days at 29°C (G) *Ras* ^*V12S35*^ over-expressing midgut (H) *Ras* ^*V12S35*^ and *cic* ^*ΔC2*^ co-over expressed midgut. Size of GFP+ clones (green) in *Ras* ^*V12S35*^ and *cic* ^*ΔC2*^ co-overexpressing midgut was significantly reduced. Samples were stained with anti-PH3 (red) and DAPI to visualize nuclei. (I-K) ISC mitoses as quantified by scoring PH3+ cells. (I) Quantification of ISCs mitoses for the *λtop* and *cic* epistasis test. The increase in mitoses induced by *λtop* was completely suppressed by *cic or cic* ^*ΔC2*^ over expression. (J) Quantification of ISC mitoses from *Ras* ^*V12S35*^/*cic* epistasis tests. The increase in mitosis induced by *Ras* ^*V12S35*^ was partially suppressed by *cic or cic* ^*ΔC2*^ over expression. (K) Quantification of ISCs mitosis in *cic* and either *Egfr or Ras* double knock down midguts. The increase in ISC mitoses induced by *cic-RNAi* is still observed when either *Egfr* or *Ras RNAi* is also expressed. Error bars represent standard deviations. Statistical significance was determined by Student’s t test (*p<0.05, **p<0.01, ***p<0.001, ****p<0.0001). Scale bars represent 50μm (A-H).

To test the function of Cic in EGFR signaling we performed epistasis tests. After 2 days of clone induction with the *esg* ^*ts*^ *F/O* system, control midguts generated only 2-cell clones, whereas clones overexpressing an activated variant of the EGFR, (*λtop*), grew very large and showed increased ISC division. However, when *cic* or *cic* ^*ΔC2*^ was co-overexpressed along with *λtop*, clone sizes and ISC mitoses were significantly reduced (Fig 2A–2C and 2I). Overexpression of Cic or Cic^ΔC2^ could also partially inhibit the ISC growth effects of *Ras* ^*V12S35*^, an activated allele that can activate RAF/MAPK signaling but not PI3K signaling [38] (Fig 2D, 2H, and 2J). Furthermore, we used *esg* ^*ts*^ to induce *Egfr-RNAi*, *or Ras-RNAi* in combination with *cic-RNAi*. The *cic*, *Egfr* or *cic*, *Ras* double RNAi animals exhibited increased ISC mitosis relative to controls expressing *Ras-RNAi* or *Egfr-RNAi* only (Fig 2E–2G and 2K), indicating that reduced ISC proliferation caused by the inactivation of EGFR signaling can be restored by Cic knock-down. These epistasis data further support the hypothesis that Cic acts as a negative downstream effector of EGFR to regulate ISC proliferation.

### EGFR signaling controls Cic subcellular localization

**Fig 3:**
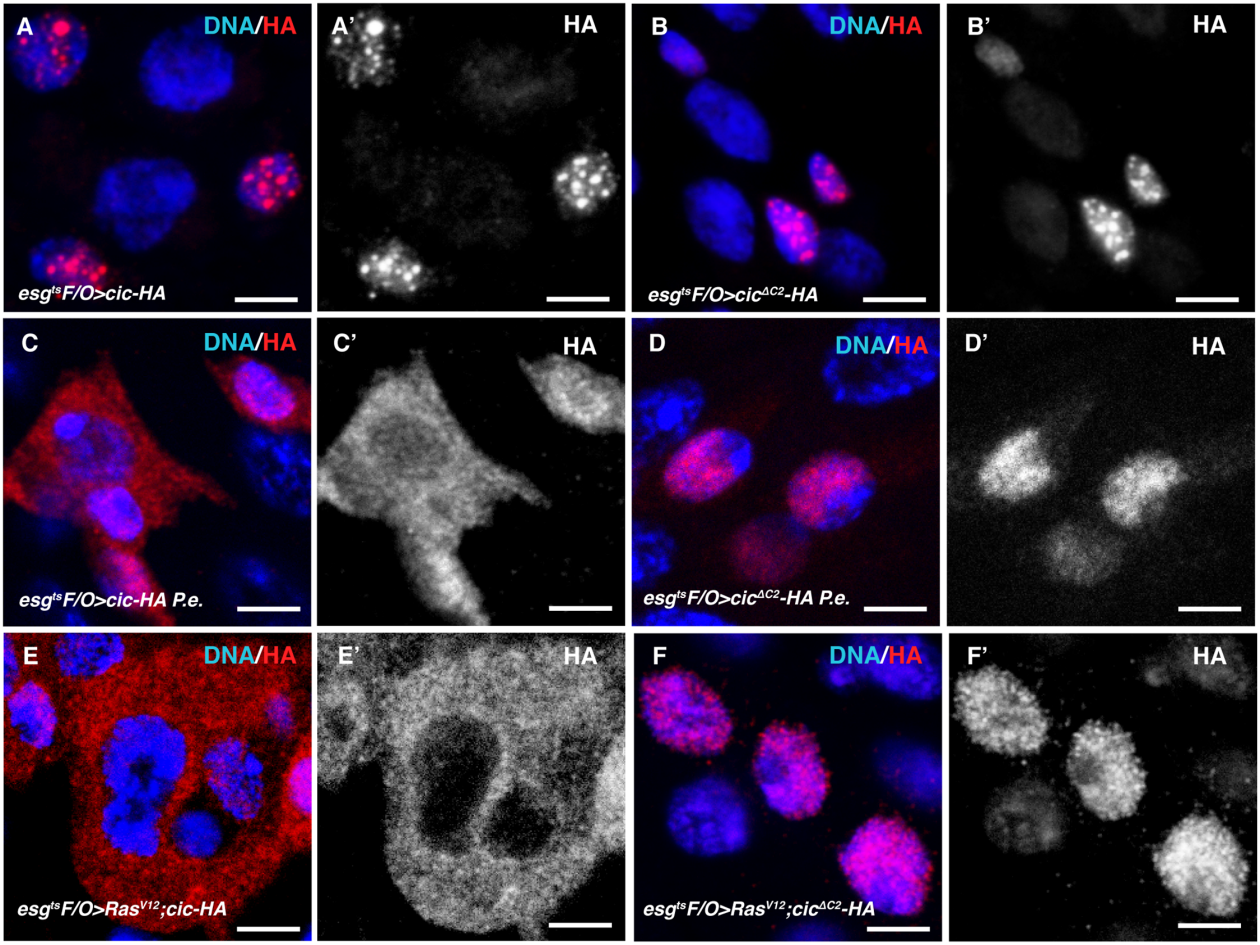
EGFR signaling controls Cic subcellular localization in ISCs. (A, B) Cic and Cic^ΔC2^ localized in the nuclei. Transgene expression was induced using the *esg* ^*ts*^ *F/O* system at 29°C for 2 days. HA-tagged Cic or Cic^ΔC2^ protein was detected by anti-HA antibody (red). Nuclear DNA is marked by DAPI staining (blue) (A) *cic-HA* overexpressing midgut. (B) *cic* ^*ΔC2*^ *-HA* overexpressing midgut. (C, D) Cic but not Cic^ΔC2^ protein accumulated in the cytoplasm after *P*.*e*. infection. (C) *cic-HA* overexpressing midgut, exposed to *P*.*e*. bacteria for 16 hours. (D) *cic* ^*ΔC2*^ *-HA* overexpressing midgut after 16 hours *P*.*e*. infection. (E, F) Cic protein accumulated in the cytoplasm when EGFR signaling was activated by *Ras* ^*V12S35*^. (E) *Ras* ^*V12S35*^ and *cic-HA* overexpressing midgut. (F) *Ras* ^*V12S35*^ and *cic* ^*ΔC2*^ *-HA* overexpressing midgut. Cic ^ΔC2^ proteins stayed in the nucleus even after overexpressing *Ras* ^*V12S35*^ to activate MAPK signaling. Scale bars represent 5μm.

To understand how EGFR signaling controls Cic in ISCs, we expressed HA-tagged Cic or Cic^ΔC2^ protein in midgut progenitor cells (ISCs and EBs). As expected, HA-tagged Cic or Cic^ΔC2^ proteins were only detected in nuclei under normal conditions (Fig 3A–3A’ and 3B–3B’). However, HA-tagged Cic protein accumulated nearly exclusively in the cytoplasm when *Ras* ^*V12S35*^ was co-expressed with it (Fig 3E–3E’). In contrast, Cic^ΔC2^ remained in the nucleus even following ectopic *Ras* ^*V12S35*^ expression (Fig 3F–3F’). A similar but milder re-localization of Cic protein from the nucleus to the cytoplasm was observed following *P*.*e*. infection (Fig 3C and 3C’), a treatment known to increase MAPK signaling in the gut [11]. It is interesting to note that Cic^ΔC2^ did not completely suppress *Ras* ^*V12S35*^ induced ISC proliferation, even though it remained localized to nuclei in *Ras* ^*V12S35*^ expressing cells (Fig 2H and 2J). However, nuclear Cic^ΔC2^ lost its characteristic punctate localization in the presence of *Ras* ^*V12S35*^ expression, and became more diffusely localized in the nucleoplasm (Fig 3F–3F’). These results suggest that, although EGFR signaling controls Cic nucleo-cytoplamic localization via the C2 motif, there may be a second MAPK-dependent mechanism to regulate Cic repressor activity, involving dissociation from chromatin, that is C2-independent.

### Cic represses cell cycle genes in ISCs

Cic has been studied in several cell types from both *Drosophila* and humans. In human melanoma cells, CIC represses mRNA expression of the PEA3 subfamily of ETS transcription factors, namely ETV1, ETV4 and ETV5 [29]. In early *Drosophila* development post-transcriptional down-regulation of Cic by the Torso and EGFR pathways regulates terminal and dorsal-ventral patterning, respectively, by allowing expression of Cic target genes such as *huckebein* (*hkb*), intermediate *neuroblasts defective* (*ind*), and *argos* (*aos*) [39]. However, a genome-wide mapping of Cic target genes has not yet been reported.

**Fig 4:**
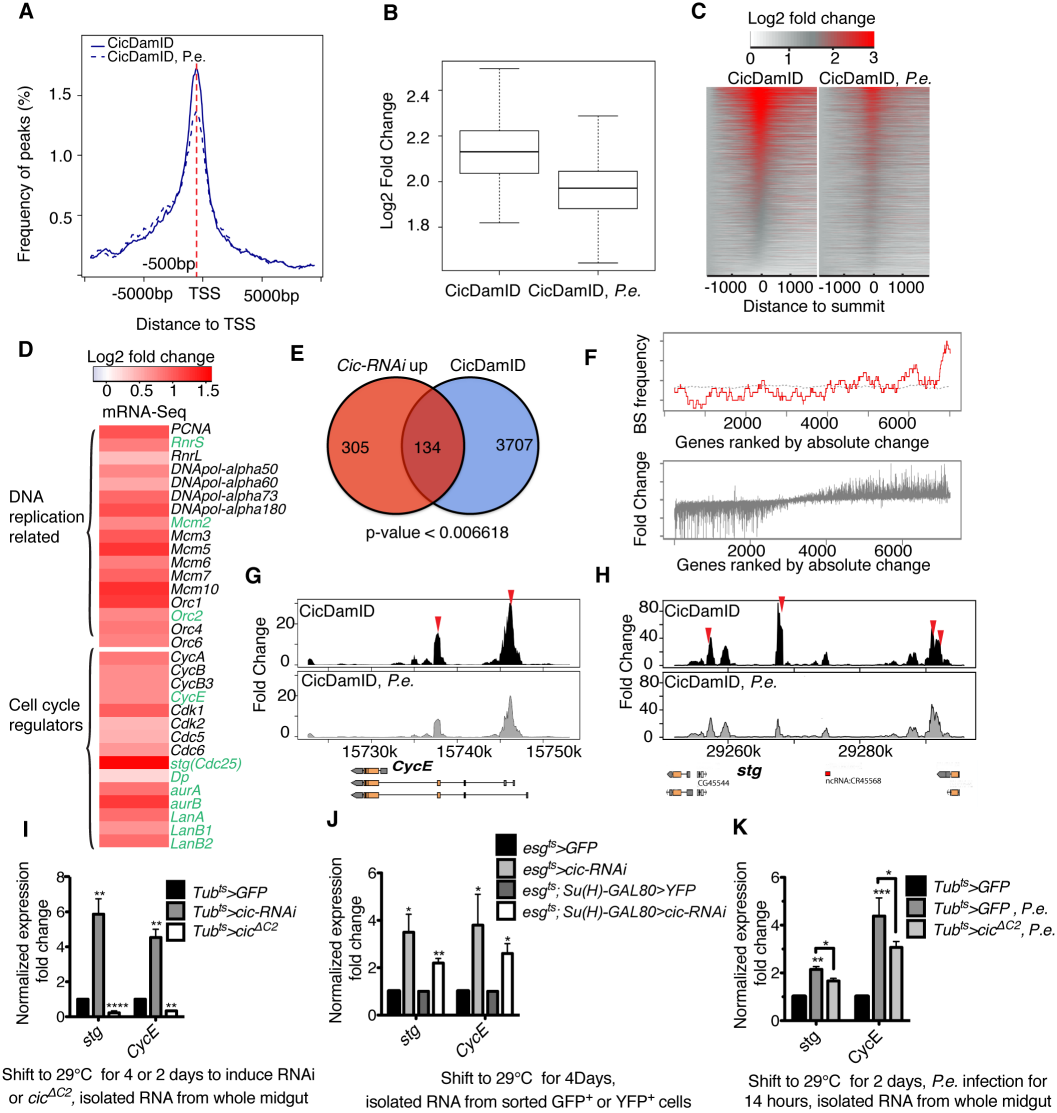
Cic targets genes in ISCs found by DamID-Seq. (A) Graph showing the location of Cic binding relative to annotated transcript TSSs. The distance is from the summit of the Cic peaks to the nearest TSS. Dashed red line showed the summit of the graph is 500bp away from TSS. (B) Box plot showing fold change of peaks in CicDamID and *P*.*e*. infected CicDam. (C) Heatmap showing fold enrichment of Cic peaks from Cic DamID-Seq without or with *P*.*e*. infection. Y axis represents genes associated with the Cic binding peaks. (D) Expression heatmap of cell cycle regulators and DNA replication related genes from RNA-Seq data from *cic-RNAi* expressing FACS sorted progenitor cells. The names of the genes that had Cic binding sites by DamID are written in green. (E) Venn diagram showing the overlap between genes upregulated > 1.5 fold upon *cic-RNAi* (left) and genes associated with Cic binding peaks (right) in ISC/EBs. (F) Graph showing correlation between genes upregulated in Cic-depleted progenitor cells, and the Cic-DamID peaks that changed significantly upon *P*.*e*. infection (upper panel). Lower panel show genes ranked by absolute expression change, and then plotted for expression fold change (bottom). (G, H) Cic binding sites in the *CycE* and *stg* loci, as determined by Cic DamID-Seq in ISC/EBs from control (above) and *P*.*e*. infected (below) midguts. Vertical bars represent the log2 ratio of the Dam-fusion signal to the Dam-only signal. Red arrows indicate TGAATG(G/A)A motifs. (I-K) mRNA level fold changes of *stg* and *CycE* analyzed by qRT-PCR and normalized to *β-Tub* and *Rp49*. (I) *stg* and *CycE* fold enrichment from whole midguts after knocking down or over expressing *cic* in all cells using the *tub* ^*ts*^ *(tubGal4; tubGal80ts)* driver. Transcription of both *stg* and *CycE* was induced in *cic* knock-down midguts and inhibitied in *cic* over-expressing midgut. (J) *stg* and *CycE* expression is upregulated in *cic*-depleted, FACS-sorted progenitor cells (ISC &EB) and ISCs. (K) *stg* and *CycE* expression fold change in *cic* over expressing midguts after *P*.*e*. infection. The induction of *stg* and *CycE* by *P*.*e*. infection was suppressed by *cic* ^*ΔC2*^ overexpression. Statistical significance was determined by Student’s t test (*p<0.05, **p<0.01, ***p<0.001, ****p<0.0001). Error bars in each graph represent standard deviations.

To identify Cic target genes involved in ISC growth and proliferation we profiled Cic binding throughout the genome using the “TaDa” (Targeted DamID)” technique. The TaDa method involves low-level expression of a GAL4-inducible Dam methylase-fusion protein in a specific cell type, enabling cell-specific profiling without cell isolation [40,41]. Here, we induced a low level of Dam-only or Dam-Cic fusion protein in progenitor cells (ISC & EB) using the *esg* ^*ts*^ system and a 24-hour induction. Genomic DNA was extracted from isolated midguts, digested with Dpn I, which cuts only methylated GATCs, and amplified. The amplified gDNA fragments were subjected to high-throughput sequencing, rather than tiling microarrays as previously reported [40]. We identified 2279 binding sites that were highly enriched (log2 fold change > 3, false discovery rate<0.01%) when comparing Dam-Cic to Dam alone samples (S1 Table). These sites were non-randomly distributed in the genome, and were significantly over-represented ~500 bp 5’ to Transcription Start Sites (TSS; Fig 4A). Cic DamID was also performed on progenitor cells from *P*.*e*. infected midguts. After a 24 hours induction of Dam or Dam-Cic transgenes via the *esg* ^*ts*^ system, flies were fed *P*.*e*. bacteria for 16 hours. The number of highly enriched (log2 fold change > 3, FDR < 0.1%) peaks was reduced to 1903. In addition, the fold change of peaks (Dam-Cic vs Dam-alone) after *P*.*e*. infection was significantly decreased (Figs 4B–4C and S4A). The frequency of peaks adjacent to TSS was also significantly reduced in the *P*.*e*.*-*infected midgut sample (Fig 4A). We believe that this decrease was due to the change of Cic localization from the nucleus to cytoplasm, which was caused by the activation of EGFR/Ras/MAPK signaling after infection.

To further understand how Cic regulates ISC proliferation we performed gene expression profiling using amplified mRNA from FACS-sorted esg+ progenitor cells that expressed *cic-RNAi*, and controls. As a way to identify potentially direct target genes of Cic, the RNA-Seq and DamID-Seq data sets were cross-compared. Amongst 439 transcriptionally up-regulated genes (>1.5 fold change, 90% CI) (S2 Table), a large fraction [134 genes, (S3 Table)] had Cic binding sites as defined by DamID (Fig 4E). We next examined the enrichment of the DamID peaks in the transcriptionally induced genes, ranked by absolute expression change in *cic* knockdown progenitor cells (see Materials and Methods). Cic binding peaks that were significantly reduced upon *P*.*e*. infection (< 2 fold change) were enriched in up-regulated genes from the RNA-Seq dataset (Fig 4F). Hence, the set of genes present in the overlapping set are likely to be direct target genes of Cic. Many cell cycle regulators and genes involved in DNA replication were upregulated in Cic-depleted progenitor cells (Fig 4D). In addition, a large portion of cell cycle control genes that were upregulated upon *cic-RNAi*, including *string (stg*, *Cdc25)* and *Cyclin E (CycE)*, had Cic binding sites (Fig 4D, 4G, and 4H). To further assess the reliability of this approach we examined the occupancy of Cic on its previously characterized direct target gene-*aos* [39]. Our DamID-Seq data showed that *aos* contained two Cic binding sites within its enhancer, and that their occupancy was significantly reduced after *P*.*e*. infection (S4B Fig). The significant induction of *aos* transcription was verified both by RNA-Seq and qRT-PCR data from FACS-sorted progenitor cells expressing *cic-RNAi* (S4C Fig).

Having confirmed the reliability of our approach for identifying genes that are repressed by Cic in ISCs, we focused on genes likely to contribute to ISC proliferation. We were interested in *stg* and *CycE* because they are transcriptionally induced in proliferating ISCs [42], required for ISC divisions, and also sufficient to induce sustained ISC division when co-overexpresssed [42]. To further test whether Cic regulates the transcription of *stg* and *CycE* we measured their normalized expression ratios in gain- or loss-of-function Cic midguts via RT-qPCR (Fig 4I–4K). The *stg* and *CycE* mRNAs were significantly increased in Cic-depleted midguts, and decreased in midguts expressing the dominant active Cic^ΔC2^. Strong inductions of *stg* and *CycE* were also observed in Cic-depleted progenitor cells or ISCs purified using FACS (Fig 4J). Moreover, both the *stg* and *CycE* loci had multiple strong Cic-Dam-ID binding peaks containing TGAATG(G/A)A motifs, and binding these peaks were reduced by *P*.*e*. infection (Fig 4G and 4H). Consistently, the induction of *stg* and *CycE* transcription upon *P*.*e*. infection was significantly repressed by Cic^ΔC2^ overexpression (Fig 4K). These data support the notion that Cic controls ISC cell cycle progression by directly repressing transcription of *stg* and *CycE* via binding sites in their regulatory regions.

### Cic represses *pnt* and *Ets21C*

**Fig 5:**
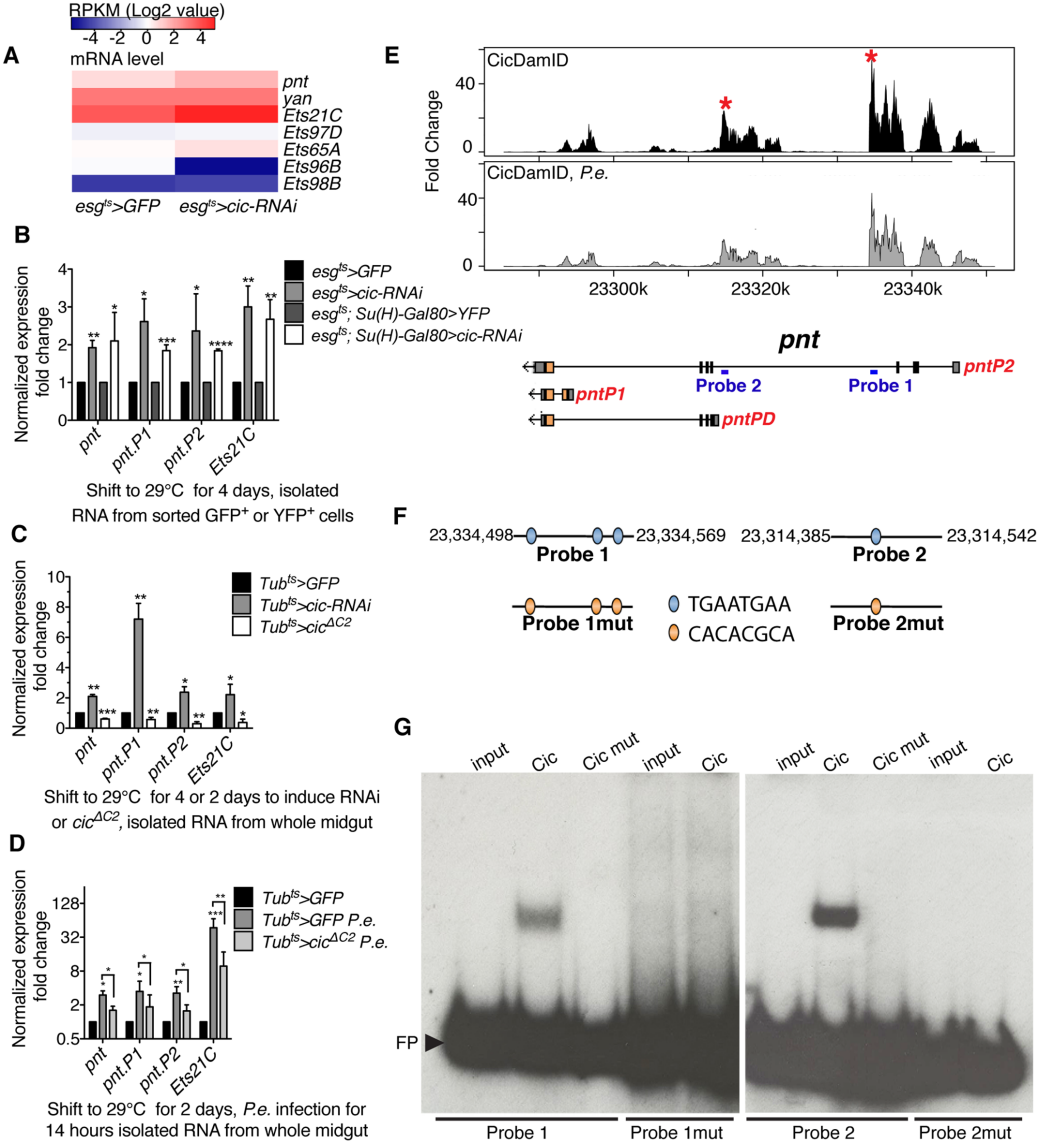
Cic regulates Pnt expression through binding to its genomic locus. (A) Heatmap of mRNA levels indicating RPKM values from RNA-Seq data from control and *cic-RNAi* expressing, FACS-sorted progenitor cells. (B-D) Relative expression of *pnt*, *pntP1*, *pntP2* and *Ets21c* as analyzed by qRT-PCR and normalized to *β-Tub* and *Rp49*. (B) Fold change of expression from the *cic* depleted FACS-sorted progenitor cells and ISCs. (C) *pnt*, *pntP1*, *pntP2* and Ets21C were upregulated in *cic* knock down midguts and downregulated in *cic* overexpressing midguts. (D) Expression change in *cic* overexpressed midgut after *P*.*e*. infection. Error bars represent standard deviation. Statistical significance was determined by Student’s t test (*p<0.05, **p<0.01, ***p<0.001, ****p<0.0001). (E) Cic binding sites in the *pnt* locus, as determined by Cic DamID-Seq of esg+ progenitor cells from control and *P*.*e*. infected midguts. Vertical bars represent the log2 ratio of the Dam-fusion signal to the Dam-only signal. Peaks also found in Cic ChIP-Seq from embryos are marked by asterisks. Positions of EMSA probes from the *pnt* locus are indicated by blue bars. (F) Diagram of probes containing TGAATGAA sites. These sites were replaced with other sequences in probes 1 and 2 to generate probes 1mut and 2mut. (G) DNA binding of Cic and HMG-box mutated Cic to probe 1 or 1mut (left panel). DNA binding of Cic and HMG-box mutated Cic to probe 2 or probe 2mut (right panel). FP indicates “free probes.”

It has been suggested that Cic might regulate the transcription of certain members in a subfamily of ETS transcription factors [29,31]. Consistent with this, we identified the *Drosophila* ETS transcription factors *pnt* and *Ets21C* as potential *Cic* direct target genes by both RNA-Seq and DamID-Seq (Figs 5 and S5). These genes contain Cic binding sites, were highly expressed in midgut progenitor cells, and were significantly induced upon infection or *cic* depletion or mutation. Notably, induction of *pnt* and *Ets21C* was detected in FACS-sorted ISCs depleted of Cic (Fig 5B). Moreover, the induction of *pnt* and *Ets21C* expression by *P*.*e*. infection was suppressed when the dominant active form, Cic^ΔC2^ was overexpressed (Fig 5D). Similar effects were observed when Cic was either depleted or overexpressed in whole midgut samples (Figs 5C and S5C). These data suggest that Cic also regulates *pnt* and *Ets21C* transcription in *Drosophila* midgut ISCs, by directly binding to these loci. As in the case of *stg* and *CycE*, this regulation appeared to be modulated by *P*.*e*. infection, most likely in a MAPK-dependent manner.

### Cic represses *pnt* via a TGAATGAA motif

The HMG box of Human Cic binds to TGAATG(G/A)A octamers *in vitro* [31]. This motif was also verified as a Cic binding sequence in several Cic target genes in *Drosophila* embryos and wing discs [39]. Notably, the TGAATG(G/A)A motif was observed in 692/2279 Cic binding sites in our DamID-Seq dataset (p-value = 3.045475× 10^−11^). Each of the four Cic target genes discussed above contained more than one TGAATG(G/A)A motifs in its Cic binding sites. Moreover, TGAATGAA motifs found in the *pnt* locus also mapped to Cic binding sites that we determined from *Drosophila* embryo ChIP-Seq (Fig 5E). This suggests that Cic may bind to the *pnt* locus via TGAATGAA octamers, and that the occupancy of Cic at the *pnt* locus may also be conserved in different *Drosophila* cell types. To further evaluate this hypothesis we performed electrophoretic mobility shift assay (EMSA). Cic showed specific binding to two DNA fragments from the *pnt* locus that were identified as prominent *in vivo* Cic binding peaks by DamID-Seq and ChiP-Seq (Fig 5F and 5G). Importantly, the EMSA interaction was lost when the HMG-box in Cic was mutated, or when the TGAATGAA motifs were mutated. These data strongly support the idea that Cic directly regulates *pnt* transcription by directly binding to TGAATGAA motif in *pnt* locus.

### Pnt regulates ISC proliferation as a direct target of Cic

**Fig 6:**
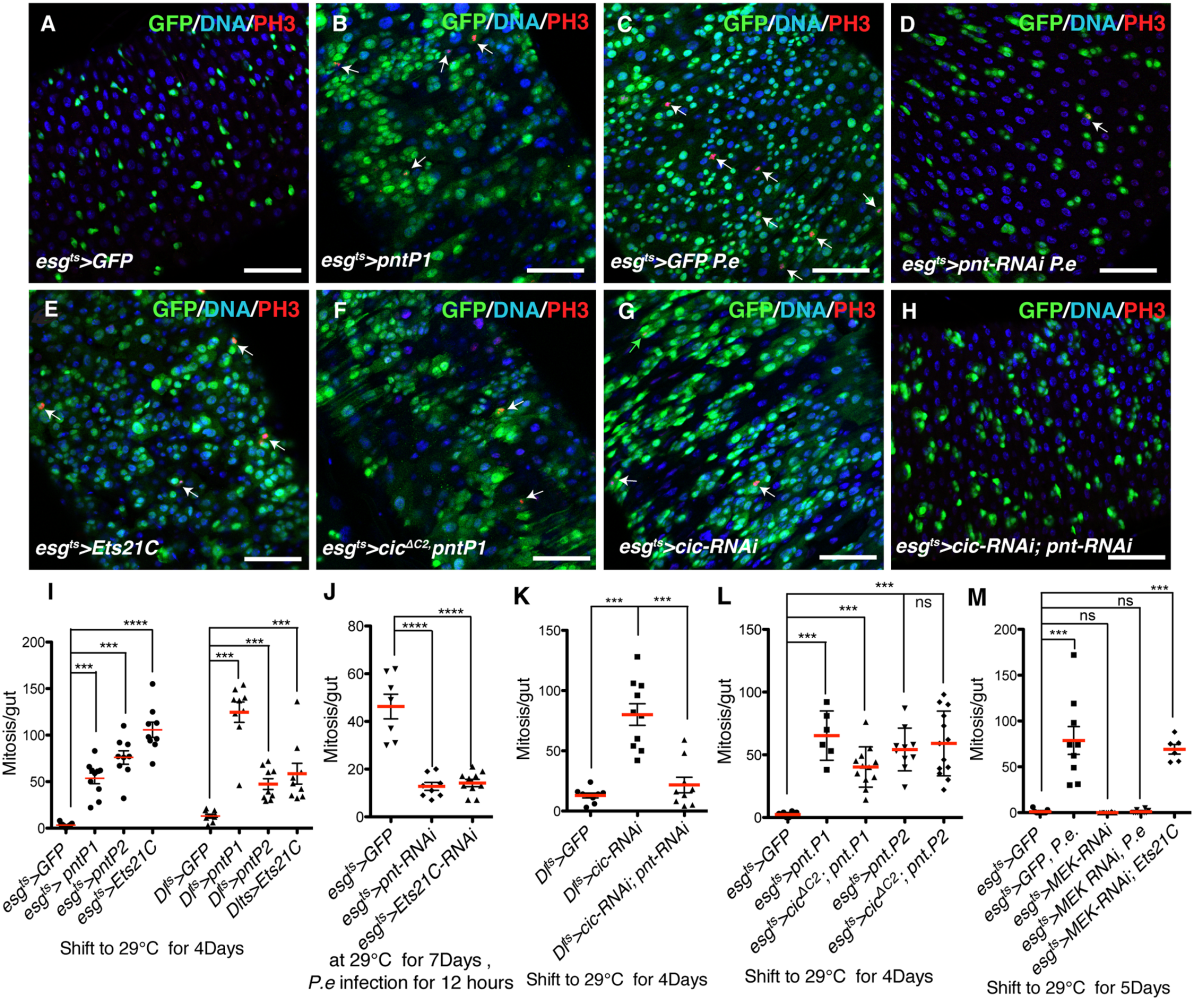
Cic controls ISC proliferation by regulating *pnt* transcription. (A–E) Effects caused by *pntP1* overexpression and RNAi’s. Transgenes were induced using the *esg* ^*ts*^ system at 29°C for 4 days, and samples were stained for GFP (green), DNA (blue) and mitoses (PH3, red). White arrows pointing out PH3 signals. (A) Control adult midgut. (B) *pntP1* overexpressing midgut after 4 days induction at 29°C. (C) Control midgut after 12 hours *P*.*e*. infection. (D) *pnt* knockdown midgut after 12 hours *P*.*e*. infection. Fewer GFP+ and PH3+ cells are observed. (E) *Ets21C* overexpressing midgut, showing more PH3+ ISCs (arrows) and GFP+ ISCs and EBs (green). (F-H) Ectopic expression or loss of *pnt* bypasses ISC phenotypes caused by *cic* overexpression or depletion. (F) *pnt* and *cic* ^*ΔC2*^ co-over-expressing midgut after 4 days induction at 29°C. GFP+ progenitor cells were still able to proliferate. (G) *cic* knockdown adult midgut and (H) *pnt*, *cic* double knockdown midgut. The increased number of progenitor cells marked by GFP upon *cic* knockdown was decreased by also knocking down *pnt*. (I-L) Quantification of PH3+ cells in adult midguts of the indicated genotypes. (I) *pntP1*, *pntP2* or *Ets21C* overexpression driven by *esg* ^*ts*^ or *Dl* ^*ts*^. All the *pntP1*, *pntP2* and *Ets21C* overexpressing midguts contained more dividing ISCs. (J) *pnt* or *Ets21C* knockdown midguts after *P*.*e*. infection. ISC mitoses caused by *P*.*e*. infection were reduced in *pnt* or *Ets21C* knockdown midguts. (K) *pnt* and *cic* knock down using *Dl* ^*ts*^ system. Fewer mitotic ISCs were observed in the *pnt* and *cic* double knockdown midgut than the *cic* knockdown midgut. (L) *pnt* and *cic* ^*ΔC2*^ co-overexpressing midguts. *cic* ^*ΔC2*^ overexpression could not inhibit ISC mitoses caused by *pnt* overexpression. (M) Quantification of PH3+ cells from adult midguts following *P*.*e*. infection. *MEK-RNAi* completely blocked infection-driven ISC mitoses, but could not inhibit ISC proliferation driven by overexpressed Ets21c. Statistical significance was determined by Student’s t test (*p<0.05, **p<0.01, ***p<0.001, ****p<0.0001). Error bars in each graph represent standard deviation. Scale bars represent 50μm.

Pnt is believed to be a downstream effector of EGFR signaling in developing *Drosophila* eyes [43,44,45]. The *pnt* locus produces two alternative transcripts that encode two different protein isoforms: PNTP1 and PNTP2 [44]. PNTP1 was proposed to be a constitutive activator of transcription, whereas PNTP2 has a PNT (pointed) domain that was reported to be phosphorylated by MAP kinase *in vitro* [45]. The mutant protein, PNTP2^T151A^, which cannot be phosphorylated *in vitro*, was unable to rescue *pnt* phenotype in eyes but instead enhanced the mutant phenotype, suggesting that the PNT domain is an auto-inhibitory domain that can be inactivated by MAPK-dependent phosphorylation [45]. Furthermore PNTP2 is thought to induce transcription of PNTP1, which might thereby encode the final nuclear effector of the EGFR pathway in eye discs [43]. In the midgut, we found an interesting interaction between Pnt and Cic: *pntP1* and *pntP2* were both induced when Cic was depleted, and both decreased when Cic was overexpressed (Figs 5B, 5C, and S5A). The expression of transcripts encoding both isoforms was also increased in *P*.*e*. infected guts (Figs 5D and S5B). This raises the possibility that *pnt* might be an important downstream effector of Cic in controlling ISC proliferation. To test this we over-expressed either *pntP1* or *pntP2* in progenitor cells using the *esg* ^*ts*^ or *Dl* ^*ts*^ driver systems. After 4 days of transgene induction a dramatic increase in ISC division was evident in response to either *pntP1* or *pntP2* (Figs 6A–6B, 6I, and S6A–S6B). Conversely, mutant clones that were generated using a *pnt* null allele (*pnt* ^*Δ88*^) [46] did not grow past the 2-cell stage (S6D Fig). Moreover, when we depleted *pnt* in progenitor cells by expressing a *pnt-RNAi* that recognizes both isoforms, or generated homozygous *pnt* null mutant ISCs via MARCM, ISC proliferation after *P*.*e*. infection was suppressed (Figs 6C–6D, 6J, and S6E). Next, we investigated the functional significance of the inhibition of *pnt* expression by Cic. Whereas loss of Cic function induced massive ISC proliferation, inhibiting both isoforms of *pnt* in this context suppressed this over-proliferation (Figs 6G–6H, 6K and S6F–S6G). Conversely, when we over-expressed either *pntP1* or *pntP2* in ISCs that also overexpressed Cic^ΔC2^, the inhibitory effect of Cic^ΔC2^ on proliferation was bypassed and the cells divided (Figs 6F, 6L and S6C). Hence, a significant fraction of the ISC over proliferation caused by Cic knockdown can be attributed to Cic’s effects on *pntP1 and pntP2*

Interestingly, mutant clones generated using a *pntP1* specific mutant allele, *pnt* ^*Δ33*^ [45,47], or a *pntP2* specific mutant allele, *pnt* ^*Δ78*^ [45,47], grew normally. However ISCs mutant for the *pnt* null allele *pnt* ^*Δ88*^ did not expand (S6D Fig). In addition, *pnt* ^*Δ33*^ and *pnt* ^*Δ78*^ homozygous clones in which *cic* was depleted by RNAi had similar numbers of cells to *cic*-depleted control clones (*i*.*e*. they overgrew), whereas *pnt* ^*Δ88*^ null mutant clones contained significantly fewer cells (S6G Fig). These data not only support our conclusion that *pnt* is required for ISC proliferation as a target of Cic, but show that PNTP1 and PNTP2 have redundant function in regulating ISC proliferation. Furthermore, *pntP2* homozygous mutant ISCs did not appear to have any defect in proliferation upon *P*.*e*. infection (S6E Fig). Overall these results indicate that *pntP2*, the isoform proposed to be activated directly by MAKP phosphorylation [45], is not specifically required in ISC proliferation.

Pnt is the *Drosophila* ortholog of the human ETS2 transcription factor and has a conserved ETS-type DNA binding domain, while Ets21C is the *Drosophila* ortholog of the human proto-oncogene ERG. In addition to having Cic binding sites, RT-PCR and RNA-Seq data showed that Ets21C was highly induced upon *P*.*e*. infection (Figs 5A and S5C). Moreover RNAi mediated depletion experiments indicated that Ets21C was also required for ISC proliferation in response to *P*.*e*. infection (Fig 6J). Over-expression of *Ets21C* caused a strong increase of ISC division (Fig 6E and 6I) suggesting that transcriptional induction of *Ets21C* could promote ISC proliferation. Furthermore, ectopic expression of *Ets21C* in progenitor cells could bypass the strong growth-suppressive effect of depleting MEK (Fig 6M). These data indicated that Cic controls ISC proliferation in part by regulating *Ets21C* transcription.

Finally, we tested whether Yan, an inhibitory ETS type transcription factor, reported to be MAPK responsive and to compete with Pointed for binding to common sites on the DNA [45,48,49], had an opposite function in ISCs. Although *yan* mRNA is expressed in the midgut (Fig 5A), *yan* depletion from ISCs did not produce a detectable effect (S6F Fig). Two independent *yan-RNAi* lines were used, both of which were proven to be effective by qRT-PCR (S6H Fig). In summary these observations suggest that EGFR signaling controls ISC growth and division by regulating the activity of Cic, Pnt and Ets21C but not Yan, and that Cic directly represses *pntP1*, *pntP2* and *Ets21C* in this context.

## Discussion

**Fig 7:**
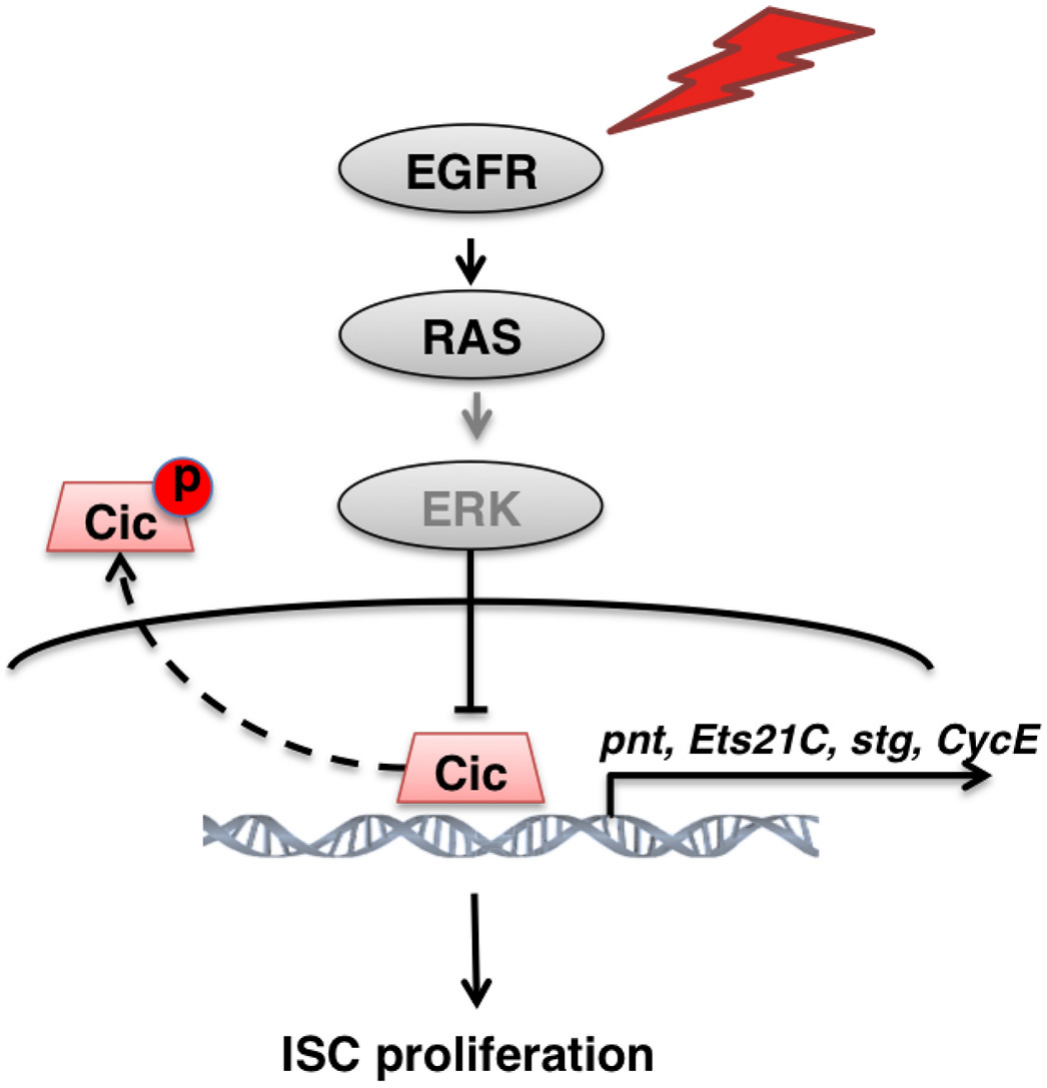
Model for Cic control of Drosophila ISC proliferation. Upon damage, activated EGFR signaling mediates activation of ERK, which phosphorylates Cic, and relocates it to the cytoplasm. As a result, *stg*, *CycE*, *Ets21C* and *pnt* transcription are relieved from Cic repression, and induce ISC proliferation.

It is well established that EGFR signaling is essential to drive ISC growth and division in the fly midgut [10,11,12]. However, the precise mechanism via which this signal transduction pathway activates ISCs has remained a matter of inference from experiments with other cell types. Moreover, despite a vast literature on the pathway and ubiquitous coverage in molecular biology textbooks, the mechanisms of action of the pathway downstream of the MAPK are not well understood for any cell type. From this study, we propose a model summarized in Fig 7. Multiple EGFR ligands and Rhomboid proteases are induced in the midgut upon epithelial damage, which results in the activation of the EGFR, RAS, RAF, MEK, and MAPK in ISCs. MAPK phosphorylates Cic in the nucleus, which causes it to dissociate from regulatory sites on its target genes and also translocate to the cytoplasm. This allows the de-repression of target genes, which may then be activated for transcription by factors already present in the ISCs. The critical Cic target genes we identified include the cell cycle regulators *stg (Cdc25)* and *Cyclin E*, which in combination are sufficient to drive dormant ISCs through S and M phases, and *pnt* and *Ets21C*, ETS-type transcriptional activators that are required and sufficient for ISC activation.

Although we found more than 2000 Cic binding sites in the ISC genome, not all of the genes associated with these sites were significantly upregulated, as assayed by RNA-Seq, upon Cic depletion. One possible explanation for this is that Cic binding sites from DamID-Seq were also associated with other types of transcription units (miRNAs, snRNAs, tRNAs, rRNAs, lncRNAs) that were not scored for activation by our RNA-Seq analysis. Indeed a survey of the Cic binding site distributions suggests this (S5 Table). This might classify some binding sites as non-mRNA-associated. However, it is also possible that many Cic target genes may require activating transcription factors that are not expressed in ISCs. Such genes might not be strongly de-repressed in the gut upon Cic depletion.

In other *Drosophila* cells MAPK phosphorylation is thought to directly inactivate the ETS domain repressor Yan, and to directly activate the ETS domain transcriptional activator Pointed P2 (PNTP2) [45,50]. In fact Pnt and Yan have been shown to compete for common DNA binding sites on their target genes [45,48,49]. Thus, previous studies proposed a model of transcriptional control by MAPK based solely on post-translational control of the activity of these ETS factors. However, we found that Pnt and Ets21C were transcriptionally induced by MAPK signaling, and could activate ISCs if overexpressed, and that depleting *yan* or *pntP2* had no detectable proliferation phenotype. In addition, overexpression of PNTP2 was sufficient to trigger ISC proliferation, suggesting either that basal MAPK activity is sufficient for its post-translational activation, or that PNTP2 phosphorylation is not obligatory for activity. Moreover, *pntP2* loss of function mutant ISC clones had no deficiency in growth (S6D Fig) even after inducing proliferation by *P*.*e*. infection, which increases MAPK signaling (S6E Fig). These observations indicate that the direct MAPK→PNTP2 phospho-activation pathway is not uniquely or specifically required for ISC proliferation. Or results suggest instead that transcriptional activation of *pnt* and *Ets21c* via MAPK-dependent loss of Cic-mediated repression is the predominant mode of downstream regulation by MAPK in midgut ISCs.

In addition to activating ISCs for division, EGFR signaling activates them for growth. Previous studies showed loss of EGFR signaling prevented ISC growth and division, and that ectopic *Ras* ^*V12*^ expression could accelerate the growth not only of ISCs but also post-mitotic enteroblasts [11]. Similarly, our study shows that loss of *cic* caused ISC clones to grow faster than controls, by increasing cell number as well as cell size (Figs 1H and S2C–S2H). For instance, increased size of GFP^+^ ISCs and EBs was observed when *cic-RNAi* was induced by the *esg* ^*ts*^ *or esg* ^*ts*^ *F/O* systems (Figs 1B, 6G and S2B). Therefore, in our search for Cic target genes we specifically checked probable growth regulatory genes such as Myc, Cyclin D, the Insulin/TOR components InR, PI3K, S6K and Rheb, Hpo pathway components, and the loci encoding rRNA, tRNAs and snRNAs. We found that Cic bound to the InR, Akt1, Rheb, Src42A and Yki loci. However, of these only InR mRNA was significantly upregulated in Cic-depleted progenitor cells (S4 Table). In surveying the non-protein coding genome, we found that Cic had binding sites in many loci encoding tRNA, snRNA, snoRNA and other non-coding RNAs (S5 Table), though not in the 28S rRNA or 5S rRNA genes (S4 Table). Due to the method we used for RNA-Seq library production, our RNA expression profiling experiments could not detect expression of these loci, and so it remains to be tested whether Cic may regulate some of those non-coding RNA’s transcription to control cell growth. It is also possible that Cic controls cell growth regulatory target genes indirectly, for instance via its targets Ets21C and Pnt, which are also strong growth promoters in the midgut (Figs 6A–6B, 6E and S6A–S6B). But given that no conclusive model can be drawn from our data regarding how Cic restrains growth, one must consider the possibility that ERK signaling stimulates cell growth via non-transcriptional mechanisms, as proposed by several studies [51,52,53,54].

The critical role of Cic as a negative regulator of cell proliferation in the fly midgut is consistent with its tumor suppressor function in mammalian cancer development (S1 Fig). Also consistent with our findings are the observations that the ETS transcription factors ETV1 and ETV5 are upregulated in sarcomas that express CIC-DUX, an oncogenic fusion protein that functions as a transcriptional activator [31], and that knockdown of CIC induces the transcription of ETV1, ETV4 and ETV5 in melanoma cells [29]. Moreover the transcriptional regulation by ETS transcription factors is important in human cancer development (S7 Fig). Their expression is induced in many tumors and cancer cell lines. For example, ERG, ETV1, and ETV4 can be upregulated in prostrate cancers [55], and ETV1 is upregulated in post gastrointestinal stromal tumors [56] and in more than 40% of melanomas [57]. In addition, the mRNA expression of these ETS genes was correlated with ERK activity in melanoma and colon cancer cell lines with activating mutations in BRAF (V600E), such that their expression decreased upon MEK inhibitor treatment [58]. Furthermore, overexpression of the oncogenic ETS proteins ERG or ETV1 in normal prostate cells can activate a Ras/MAPK-dependent gene expression program in the absence of ERK activation [59]. These cancer studies imply that there is an unknown factor that links Ras/Mapk activity to the expression of ETS factors, and that some of the human ETS factors might act without MAPK phosphorylation, as does *Drosophila* PntP1. Combining our knowledge of Cic with what was previously known about CIC in tumor development, we propose that CIC is the unknown factor that regulates ETS transcription factors in Ras/MAKP-activated human tumors.

In summary, our study has elucidated a mechanism wherein Cic controls the expression of the cell cycle regulators *stg (Cdc25)* and *Cyclin E*, along with the Ets transcription factor Pnt, and perhaps also Ets21C, by directly binding to regulatory sites in their promoters and introns. Using genetic tests we show that these interactions are meaningful for regulating stem cell proliferation. Therefore, we suggest that human CIC may also lead to the transcriptional induction of cell cycle genes and ETS transcription factors in RAS/MAPK activated- or loss-of-function-CIC tumors such as brain or colorectal cancers.

## Materials and Methods

### *Drosophila* stocks and transgenes

*esg* ^*ts*^: *esg-Gal4/Cyo; tubGal80* ^*ts*^ *UAS-GFP/TM6B* [60]

*esg* ^*ts*^ *F/O*: *esg-Gal4 tubGal80* ^*ts*^ *UAS-GFP/Cyo;UASflp>CD2>Gal4/TM6B* [8]

*Tub* ^*ts*^: *tub-Gal4; tubGal80* ^*ts*^ */TM3*, *ser* [61](provided from Valeria Cavaliere lab)

*esg* ^*ts*^ *; Su(H)-Gal80*: *esg-Gal4-UAS-2XEYFP; Su(H)GBE- Gal80*, *tub-Gal80* ^*ts*^ (Gift from Steven Hou’s lab)

*UAS-λTOP/FM7* [37]

*UAS-RAS* ^*v12s35*^ [38]

*UAS-Ras RNAi* [11]

*UAS-Egfr RNAi* [11]

*UAS-cic-RNAi/Cyo* (VDRC KK103805)

*UAS-cic-RNAi/Cyo* (VDRC KK103012)

*UAS-pnt*.*P1* (Bloomington Drosophila Stock Center 869)

*UAS-pnt*.*P2* (Bloomington Drosophila Stock Center 399)

*UAS-pnt-RNAi* (Bloomington Drosophila Stock Center 31936)

*UAS-pnt-RNAi* (Bloomington Drosophila Stock Center 35808)

*UAS-yan-RNAi* (Bloomington Drosophila Stock Center 26759)

*UAS-yan-RNAi* (Bloomington Drosophila Stock Center 34909)

*UAS-yan-RNAi* (Bloomington Drosophila Stock Center 35404)

*UAS-Ets21C-RNAi* (VDRC KK103211)

*FRT82B cic* ^*fetu6*^ */ TM3*, *Sb*, *Se* (gift from Jimenez lab, Barcelona)

*w; cic* ^*fetT6*^ */TM3*, *Ser* (gift from Nilson lab, Canada)

*w; cic* ^*fetE11*^ */ TM6b* (gift from Nilson lab, Canada)

*w;* +*; UAS*-*cic*-*HA*

*w; UAS*-*cic*-*HA;* +

*w;* +*;UAS*-*cic* ^*ΔC2*^-*HA*

*w; UAS*-*cic* ^*ΔC2*^-*HA;* +

*FRT82B pnt* ^*Δ33*^ [45,47] (gift from Joseph Bateman lab, Wolfson Centre for Age-Related Diseases)

*FRT82B pnt* ^*Δ78*^ [45,47] (gift from Joseph Bateman lab, Wolfson Centre for Age-Related Diseases)

*FRT82B pnt* ^*Δ88*^[45,47] (gift from Joseph Bateman lab, Wolfson Centre for Age-Related Diseases)

### Generation of transgenic flies

The *cic* ^*ΔC2*^ was amplified from the *pCasper4—cic* ^*ΔC2*^ plasmid. The *cic* or *cic* ^*ΔC2*^ cDNAs were inserted into pUASg-attB-HA [62] vector and used to generate transgenic flies. To generate *UAS-cicDam* transgenic flies, Cic was amplified from a cDNA library prepared from midgut. This *cic* cDNA was inserted into the pUASTattB-LT3-NDam plasmid (from Andrea brand lab), and transgenics were produced.

### Ectopic expression

Ectopic expression of transgenes in the adult midgut was achieved using the temperature sensitive inducible UAS-Gal4 system [63], TARGET. Crosses were set up and maintained at 18°C, the permissive temperature. 3–7 day old flies were shifted to 29°C for different times as indicated.

### Bacterial infection

Gut infections were performed by feeding flies live *P*.*e*. in 5% sucrose on Whatman filter paper and yeast paste at 29°C.

### Clonal analysis

The MARCM system was used to generate ISC clones. In order to reduce heat shock dependent stress, the clones were induced by heat shocking 3–5 days old flies at 34°C for 20 minutes. The heat shocked flies were then kept at 25°C. Clone size was measured after 10, 20, 30 days of clone induction. The size of the clones was quantified by Fiji software measuring GFP^+^ area from z-projected confocal microscopy images.

### Immunohistochemistry and microscopy

Female adult flies were dissected in 1×PBS. Midguts were fixed in 1×PBS with 4% paraformaldehyde for 30 minutes at room temperature. Samples were washed in PBS with 0.1% X-100 (PBST) for 3x10 minutes each. Then the tissues were blocked in PBS with 0.1% X-100, 2.5%BSA, 10% NGS for at least 30 min at room temperature. All samples were incubated with primary antibody overnight at the following dilutions: rat anti-HA (1:200; Roche), guinea pig anti-Cic (1:1000, generated by author), rabbit anti-PH3 (1:1000, Millipore). After washing 3 times 10 minutes each in PBST, samples were incubated with secondary antibodies for at least 2 hours at room temperature at a dilution of 1:1000. DNA was visualized with DAPI (0.1mg/ml, Sigma), diluted 1:200. Images of Figs 1A–1B and 2E–2H were acquired by Delta vision microscope and the rest of the fluorescence images were taken by Leica SP5 confocal microscope. Images were then processed using Fiiji and Adobe Photoshop software.

### RT-qPCR

RNA was extracted from 10–12 female midguts using the RNAeasy kit (QIAGEN). RNA isolation from sorted cells was performed as previously described [64] and 100ng RNA (non-amplifed) used for reverse transcription. cDNA was synthesized by QuantiTect reverse transcription kit (QIAGEN). RT-qPCR was performed on a Light Cycler 480 II using SYBR Green I (Roche). Experiments were performed in biological triplicate. Relative fold differences in expression level of target genes were calculated as ratios to the mean of the reference genes rp49 [65] and tubulin [23]. Primer sequences are given in Supplementary Material and Methods.

### RNA-Seq and data analysis

RNA isolation and amplification from sorted cells was performed as previously described [64]. Four independent biological replicates were used for sequencing. Raw reads were checked for quality using Fastqc and subsequently aligned using Tophat2, version 2.0.9, against the Flybase genome version 6. Mapped reads were counted using HTSeq-count version 0.5.4p5 [66] with mode „union“. Genes showing a cpm value below 1 in four samples per treatment were considered as poorly expressed and filtered out before conducting differential expression analysis using edgeR, version 3.2.4 [67]. Since our replicates were generated independently, we used a paired design and corrected the resulting *p*-values by the Benjamini-Hochberg method [68]. Subsequently, genes with a fold change of 1.5 and an adjusted *p*-value lower than 0.1 were considered as significantly deregulated.

#### DamID-Seq

*UAS-Dam* and *UAS-cicDam* transgenes were induced in esg+ cells for 24 hours at 29°C and 80 guts were dissected. Genomic DNA was extracted and methylated DNA was processed and amplified as previously described [69]. Sequencing libraries were prepared according to the protocol from Andrea Brand lab (personal communication), with the following modifications. Amplified DNA from experimental and Dam-only controls was fragmented in a Covaris-S2 then digested with Sau3AI to remove the adaptors. The Truseq DNA PCR-Free Sample Preparation kit (illumina) was used to prepare the sequencing library. The library was sent for Hiseq-2000 single-end 50bp sequencing.

#### DamID-Seq analysis

Raw reads were mapped to the *Drosophlia* genome (version 6.02, www.flybase.org) using Bowtie 2 [70] with default setting. After mapping, the uniquely mapped reads were extended 300 base pair (bp) toward 3' prime, and the genome were segmented with a unit of 75 bp window. Then the number of reads falling into each window was quantified. Normalization factors were computed based on the assumption that the mean log ratio between two experiments is equal to 1. The log ratio between treatment and background was observed to follow a normal distribution; the mean and standard deviation were estimated by using the fitdistr function in R (www.r-project.org). The statistical significance of the enrichment was then computed using the estimated mean and standard deviation. Using a sliding window approach, a binding site was called, where at least 4 continuous units (4x75bp windows) had a significant enrichment (*p-*value<0.01). The false discovery rate of the binding sites was calculated similarly to the previous publication [71].

Annotation of the peaks was carried out using two approaches. The first is based on the summit of the peak. The distance between the summit and the closest gene transcription start site (TSS) was computed with the gene orientation in consideration, and the closest gene was then assigned to the peak. The second approach is based on the entire peak. If the gene is found to be overlapping with the peak, then the gene is associated with the peak.

“TGAATG[AG]A” was searched in the peaks. The total number of occurrences was quantified, as well as the number of peaks that contained the searched pattern. In order to estimate the significance of the pattern, the background was generated by randomly moving the peaks in the genome for 1000 times. The occurrence of the pattern in the random sequences was then fitted to a Negative Binomial distribution by using the fitdistr function in R (www.r-project.org). The *p*-value was computed using the pnbinom function in R (www.r-project.org). To assess the association between the RNA-Seq and DamID-Seq hits, genome wide genes were ranked by fold change or absolute expression change, and corresponding number of genes which has binding sites were calculated by a moving sum (window size = 500). The absolute change is defined as the treatment value minus the background value.

#### *in vitro* DNA binding assays

EMSA experiments were conducted using derivatives of Cic^mini^, a minimal Cic protein that is functional in the embryo [25]. Wild-type and HMG-box mutant products were synthesized with the TNT T7 Quick Coupled Transcription/Translation System (Promega); the HMG-box mutant construct lacks the peptide sequence ILGEWW. DNA probes were amplified by PCR using primers carrying Not I restriction sites, digested with Not I and end-labeled with ^32^P-dCTP and Klenow Fragment, exo- (Thermo Scientific). Binding reactions were carried out in a total volume of 20 ml containing 60 mM Hepes pH 7.9, 20 mM Tris-HCl pH 7.9, 300 mM KCl, 5 mM EDTA, 5 mM DTT, 12% glycerol, ~1 ng probe, 1 mg poly (dI-dC), 1 mg BSA and 1 ml of programmed or non-programmed (control) TNT lysate. After incubation for 20 min on ice, complexes were resolved on 5% non-denaturing polyacrylamide gels run in 0.5X TBE at 4°C, and visualized by autoradiography.

### RT-qPCR primers

Rp49 –Forward: TCGATATGCTAAGCTGTC

Rp49 –Reverse: GGCATCAGATACTGTCCCTTG

β-tubulin-Forward: ACATCCCGCCCCGTGGTC

β-tubulin-Reverse: AGAAAGCCTTGCGCCTGAACATAG

pnt-Forward: ACGCCCTATGATGCTCAATC

pnt-Reverse: TATCCAGACCCAAGGTGCTC

pntP1-Forward: CGACTGCGAACAATCTGGT

pntP1-Reverse: TTGCTGGTGTTGTAGCCTGT

pntP2-Forward: TTAGCCAATTGAACGGCATC

pntP2-Reverse: GCACAGATCCTTGCATCCAT

Ets21C-Forward: CCGGGCACTCAGGTACTACT

Ets21C-Reverse: CATACTGGAGGCCGGATCT

aos-Forward: AGAACCCATGGCTTACATGC

aos-Reverse: CGTCGCGGGTGTTAAGTTAC

yan-Forward: CTGCTGGTCATCGTGCTTAG

yan-Reverse: GACCTCAGTGTGAGCAGCAA

stg-Forward: CAGCATGGATTGCAATATCAGTA

stg-Reverse: CAACGTCGTCGTCGTAGAAC

CycE-Forward: ACAAATTTGGCCTGGGACTA

CycE-Reverse: GGCCATAAGCACTTCGTC

## Supporting Information

S1 Fig Summary of cross-cancer genetic aberrations for human CIC. The figure was reproduced from the cBioPortal for Cancer Genomics web page and modified to show only cancers with >3.3% alteration frequency. Asterisks mark colorectal cancer data from Genentech [35].
(TIF)

S2 Fig Cic regulates ISC proliferation. (A, B) RNAi-mediated depletion of Cic in ISCs and EBs using the *esg* ^*ts*^ system. *esg*+ progenitor cells (green), PH3+ (red) nuclear DNA (blue). (A) Control adult midgut (B) Cic knock down midgut after 4 days induction 29°C. Scale bars represent 50μm. (C-H) Cic mutant clones were analyzed using the MARCM system. ISC clones (green), DNA stained with DAPI (blue). Control (C, E, G) and mutant (D, F, H) ISC clones were induced with the MARCM system and examined 10 days, 20 days and 30 days later. Mutant ISCs divided faster and generated bigger clones. Scale bars represent 100μm.
(TIF)

S3 Fig Cic function has both cell autonomous and non-cell autonomous effects on ISC proliferation. (A, B) RNAi-mediated depletion of Cic in ISCs using the *Dl* ^*ts*^ system. ISCs are marked by GFP (green). Sample was also stained with anti-PH3 to detect mitoeses (red) and DAPI to detect nuclear DNA (blue). (A) Control adult midgut, (B) Cic depleted midgut after 4 days induction at 29°C. Dramatic increases in the number of GFP positive cells were observed in *cic* depleted midguts, as was large increase in ISC mitoses. (C, D) RNAi-mediated depletion of Cic in EBs using the *Su(H)* ^*ts*^ system. EB cells are marked by GFP (green). Samples were also stained with anti-PH3 (red) and DAPI (blue). (C) Control adult midgut (D) Cic depleted midgut after 4 days induction 29°C. Increases in the number of GFP positive cells and mitoses were observed in *cic* knockdown midguts. (E) Midguts as in A-C were scored for PH3+ cells after 4 days of induction of *cic-RNAi* in ISCs or EBs. (F) After 4 days induction of *cic-RNAi* in ISCs or EBs, midguts were scored for GFP+ or GFP- mitotic cells. Most mitotic cells were GFP+ when *cic-RNAi* was induced in ISCs using the *Dl* ^*ts*^ system, whereas in midguts in which *cic* was depleted in EBs, most of the mitotic cells were GFP- and likely ISCs. This indicates a non-cell autonomous effect. (G) Midguts were scored for PH3+ cells after 4 days of induction of *cic-RNAi* using the *esg* ^*ts*^ system, which targets gene expression to ISCs and EBs. Dramatic increases in the number of GFP positive cells were observed in *cic* knockdown midguts as was a large increase in ISC mitoses. Statistical significance was determined by Student’s t test (*p<0.05, **p<0.01, ***p<0.001, ****p<0.0001). Error bars in each graph represent standard deviation. Scale bars represent 20μm.
(TIF)

S4 Fig Identification of Cic direct target genes in ISCs. (A) Graph showing fold change of peaks from Cic-DamID and *P*.*e*. infected Cic-DamID samples. (B) Cic binding sites in the *aos* locus from Cic-DamID-Seq using midgut ISCs. The black peaks are from control animals, and the grey peaks are from *P*.*e*. infected animals. Plot represents the log2 ratio between the Dam-fusion signal and the Dam-only signal. Red arrows point out TGAATG(G/A)A motifs. The *aos* transcription unit is shown below the graph. Yellow boxed regions indicate the ORF. (C) mRNA expression ratio change of *aos* was analyzed by qRT-PCR and normalized to *β-Tub* and *Rp49* with non-amplified mRNA from FACS-sorted progenitor cells.
(TIF)

S5 Fig Cic directly regulates *Ets21C* and *pnt*. (A) mRNA expression heatmap of Ets transcription factors, showing fold change inductions from RNA-Seq data from whole midguts upon 6 hours *P*.*e*. infection. (B) Cic binding sites in the *Ets21C* locus from Cic-DamID-Seq from esg+ cells. Black peaks are from control samples and grey peaks are from *P*.*e*. infected midguts. The Y-axis represents the log2 ratio of the Cic-Dam fusion signal to the Dam-only signal. Red arrows point out TGAATG(G/A)A motifs. (C) Normalized mRNA expression fold change of *pnt*, *pntP1*, *pntP2* and *Ets21C* in *cic* transheterozygous mutant midguts.
(TIF)

S6 Fig Midgut functions of *pntP1*, *pntP2*, and *yan*. (A–C) Effect of *pntP2* overexpression on ISC proliferation. Transgene expression was induced using the *esg* ^*ts*^ system at 29°C for 4 days. Samples were stained with anti-GFP (green), anti-PH3(red) and DAPI (blue) to mark DNA. (A) Control adult midgut. (B) *pntP2* overexpressing midgut. The *pntP2* over expressing midgut had more GFP+ ISCs and EBs (green). (C) *pntP2* and *cic* ^*ΔC2*^ over expressing midgut. GFP positive progenitor cells were still able to proliferate in the *pnt*, *cic* ^*ΔC2*^ over-expressing midgut. (D) *pnt* mutant clones analyzed by the MARCM system. The size of the clones was quantified by counting cell numbers per clone. *pnt* ^*Δ33*^ is a *pntP1* specific mutant allele, *pnt* ^*Δ78*^ is *pntP2* specific mutant allele and *pnt* ^*Δ88*^ is *pnt* null mutant allele that affect both isoforms. Only the *pnt* ^*Δ88*^ detectably suppressed clone expansion. (E) Mitotic ratio of the *pnt* mutant clones was scored by calculating the average number of mitoses in each clone. (F) Quantification of ISC mitoses (PH3 positive cells) in *pnt* and *cic* depleted midguts or *yan* depleted midguts, using *esg* ^*ts*^ system. Fewer mitotic ISCs were observed in the *pnt* and *cic* double knock down midgut than in the *cic* knockdown midguts, showing that *pnt* is required downstream of *cic*. Yan depletion had no effect on ISC proliferation. (G) *pnt* mutant clones were generated in a *cic* depleted background using the MARCM system. The size of the clones was quantified by counting cell numbers per clone. Only the *pnt* ^*Δ88*^ null allele suppressed the growth of *cic*-depleted ISC cell clones. (H) *yan* expression ratio as measured by qRT-PCR in *yan*-depleted midguts, using two different *yan-RNAi* lines. Statistical significance was determined by Student’s t test (*p<0.05, **p<0.01, ***p<0.001, ****p<0.0001). Error bars in each graph represent standard deviation. Scale bars represent 50μm.
(TIF)

S7 Fig Summary of cross-cancer genetic aberrations for human ETS transcription factors. The figure was reproduced from the cBioPortal for Cancer Genomics web page and modified to show only cancers with >3.3% alteration frequency. (A) Cross-cancer alteration summary for EGR (the human orthologs of *Drosophila* Ets21C). (B) Cross-cancer alteration summary for EGR (the human orthologs of *Drosophila* Pnt).
(TIF)

S1 Table Cic binding peaks from Cic DamID-seq and *P*.*e*. infected Cic DamID-seq. Two lists of peaks from Cic DamID-seq and Cic DamID-Seq upon infection are included in this table. Each data sheet presents the specific genomic location of the peaks with detailed information for the Cic binding peaks such as, chromosome, Peak starting sites, Peak ending sites, Log2 fold change of CicDam/Dam-only and Summit of the peaks. (S1-1) Cic binding peaks from Cic DamID-Seq. (S1-2) Cic binding peaks from Cic DamID-Seq upon *P*.*e*. infection.
(XLSX)

S2 Table Genes differentially regulated (>1.5-fold, at 90% confidence) upon *cic-RNAi*. The table included the full list of genes whose mRNA expression was significantly changed (>1.5-fold, at 90% confidence) in sorted *esg* ^*ts*^ *UAS-GFP* cells expressing *cic-RNAi* for 4 days. Listed data include Flybase gene ID, gene symbol, Log2-fold-change for each gene as well as significance (*p*-value with Benjamini-Hochberg correction).
(XLSX)

S3 Table List of genes scored as Cic direct targets in progenitor cells. List of Flybase gene IDs for genes that were both upregulated upon *cic-RNAi*, and also associated with one or more Cic binding peak from the Cic DamID-Seq analysis. In addition, the normalized Log2 fold change of the peaks following *P*.*e*. infection, associated with the genes was also shown in the table. Some of the peaks disappeared after *P*.*e*. infection, so they were marked as #N/D (not detected). Derived from the data in S1 and S2 Tables.
(XLSX)

S4 Table Potential growth-regulatory targets of Cic, identified by RNA-Seq and DamID-Seq. Information of well-known growth promoters was listed in the table with their log2 Fold change in RNA-Seq and significance (*p*-value with Benjamini-Hochberg correction) and numbers of Cic Dam-ID peaks, found in their introns or within 5kb range of the transcription start site. Genes that have Cic binding sites are shaded grey.
(PDF)

S5 Table Cic binding to non-protein coding RNA loci. Three Lists of the non-protein coding RNAs such as tRNA, snRNA & snoRNA and non-protein coding genes that have Cic binding sites in their loci within the 5 kb range in the transcription start site were included in this table. Each data sheet shows the specific genomic location of each peak with detailed information for the Cic binding peaks such as, chromosome, Peak starting sites, Peak ending sites, Log2 fold change of CicDam/Dam-only, FlyBase ID and Gene symbol. (S5-1) Cic binding tRNAs. (S5-2) Cic binding snRNAs & snoRNAs. (S5-3) Cic binding non-protein coding genes.
(XLSX)

## References

[1] Herbst RS (2004) Review of epidermal growth factor receptor biology. International journal of radiation oncology, biology, physics 59: 21–26. 10.1016/j.ijrobp.2003.11.041 15142631

[2] Normanno N, De Luca A, Bianco C, Strizzi L, Mancino M, et al (2006) Epidermal growth factor receptor (EGFR) signaling in cancer. Gene 366: 2–16. 16377102 10.1016/j.gene.2005.10.018

[3] Krasinskas AM (2011) EGFR Signaling in Colorectal Carcinoma. Pathology research international 2011: 932932 10.4061/2011/932932 21403829 PMC3042643

[4] Downward J (2003) Targeting RAS signalling pathways in cancer therapy. Nature reviews Cancer 3: 11–22. 12509763 10.1038/nrc969

[5] Radtke F, Clevers H (2005) Self-renewal and cancer of the gut: two sides of a coin. Science 307: 1904–1909. 15790842 10.1126/science.1104815

[6] Amcheslavsky A, Jiang J, Ip YT (2009) Tissue damage-induced intestinal stem cell division in Drosophila. Cell stem cell 4: 49–61. 10.1016/j.stem.2008.10.016 19128792 PMC2659574

[7] Buchon N, Broderick NA, Poidevin M, Pradervand S, Lemaitre B (2009) Drosophila intestinal response to bacterial infection: activation of host defense and stem cell proliferation. Cell host & microbe 5: 200–211. 19218090 10.1016/j.chom.2009.01.003

[8] Jiang H, Patel PH, Kohlmaier A, Grenley MO, McEwen DG, et al (2009) Cytokine/Jak/Stat signaling mediates regeneration and homeostasis in the Drosophila midgut. Cell 137: 1343–1355. 10.1016/j.cell.2009.05.014 19563763 PMC2753793

[9] Jiang H, Edgar BA (2009) EGFR signaling regulates the proliferation of Drosophila adult midgut progenitors. Development 136: 483–493. 10.1242/dev.026955 19141677 PMC2687592

[10] Buchon N, Broderick NA, Kuraishi T, Lemaitre B (2010) Drosophila EGFR pathway coordinates stem cell proliferation and gut remodeling following infection. BMC biology 8: 152 10.1186/1741-7007-8-152 21176204 PMC3022776

[11] Jiang H, Grenley MO, Bravo MJ, Blumhagen RZ, Edgar BA (2011) EGFR/Ras/MAPK signaling mediates adult midgut epithelial homeostasis and regeneration in Drosophila. Cell stem cell 8: 84–95. 10.1016/j.stem.2010.11.026 21167805 PMC3021119

[12] Xu N, Wang SQ, Tan D, Gao Y, Lin G, et al (2011) EGFR, Wingless and JAK/STAT signaling cooperatively maintain Drosophila intestinal stem cells. Developmental biology 354: 31–43. 10.1016/j.ydbio.2011.03.018 21440535

[13] Jiang H, Edgar BA (2012) Intestinal stem cell function in Drosophila and mice. Current opinion in genetics & development 22: 354–360. 22608824 10.1016/j.gde.2012.04.002 PMC3426656

[14] Sato T, Vries RG, Snippert HJ, van de Wetering M, Barker N, et al (2009) Single Lgr5 stem cells build crypt-villus structures in vitro without a mesenchymal niche. Nature 459: 262–265. 10.1038/nature07935 19329995

[15] Sato T, van Es JH, Snippert HJ, Stange DE, Vries RG, et al (2011) Paneth cells constitute the niche for Lgr5 stem cells in intestinal crypts. Nature 469: 415–418. 10.1038/nature09637 21113151 PMC3547360

[16] Wong VW, Stange DE, Page ME, Buczacki S, Wabik A, et al (2012) Lrig1 controls intestinal stem-cell homeostasis by negative regulation of ErbB signalling. Nature cell biology 14: 401–408. 10.1038/ncb2464 22388892 PMC3378643

[17] Roberts RB, Min L, Washington MK, Olsen SJ, Settle SH, et al (2002) Importance of epidermal growth factor receptor signaling in establishment of adenomas and maintenance of carcinomas during intestinal tumorigenesis. Proceedings of the National Academy of Sciences of the United States of America 99: 1521–1526. 11818567 10.1073/pnas.032678499 PMC122223

[18] Weinberg R (2014) The Biology of Cancer: Garland Science.

[19] Harvey Lodish A B, Kaiser Chris A., Monty Krieger, Anthony Bretscher, Hidde Ploegh, Angelika Amon, Scott Matthew P. (2012) Molecular Cell Biology: W. H. Freeman

[20] Tseng AS, Tapon N, Kanda H, Cigizoglu S, Edelmann L, et al (2007) Capicua regulates cell proliferation downstream of the receptor tyrosine kinase/ras signaling pathway. Current biology: CB 17: 728–733. 17398096 10.1016/j.cub.2007.03.023 PMC2699483

[21] Jimenez G, Guichet A, Ephrussi A, Casanova J (2000) Relief of gene repression by torso RTK signaling: role of capicua in Drosophila terminal and dorsoventral patterning. Genes & development 14: 224–231. 10652276 PMC316342

[22] Goff DJ, Nilson LA, Morisato D (2001) Establishment of dorsal-ventral polarity of the Drosophila egg requires capicua action in ovarian follicle cells. Development 128: 4553–4562. 11714680 10.1242/dev.128.22.4553

[23] Krivy K, Bradley-Gill MR, Moon NS (2013) Capicua regulates proliferation and survival of RB-deficient cells in Drosophila. Biology open 2: 183–190. 10.1242/bio.20123277 23429853 PMC3575652

[24] Jimenez G, Shvartsman SY, Paroush Z (2012) The Capicua repressor—a general sensor of RTK signaling in development and disease. Journal of cell science 125: 1383–1391. 10.1242/jcs.092965 22526417 PMC3336375

[25] Astigarraga S, Grossman R, Diaz-Delfin J, Caelles C, Paroush Z, et al (2007) A MAPK docking site is critical for downregulation of Capicua by Torso and EGFR RTK signaling. The EMBO journal 26: 668–677. 17255944 10.1038/sj.emboj.7601532 PMC1794389

[26] Grimm O, Sanchez Zini V, Kim Y, Casanova J, Shvartsman SY, et al (2012) Torso RTK controls Capicua degradation by changing its subcellular localization. Development 139: 3962–3968. 10.1242/dev.084327 23048183 PMC3472588

[27] Roch F, Jimenez G, Casanova J (2002) EGFR signalling inhibits Capicua-dependent repression during specification of Drosophila wing veins. Development 129: 993–1002. 11861482 10.1242/dev.129.4.993

[28] Lim B, Samper N, Lu H, Rushlow C, Jimenez G, et al (2013) Kinetics of gene derepression by ERK signaling. Proceedings of the National Academy of Sciences of the United States of America 110: 10330–10335. 10.1073/pnas.1303635110 23733957 PMC3690897

[29] Dissanayake K, Toth R, Blakey J, Olsson O, Campbell DG, et al (2011) ERK/p90(RSK)/14-3-3 signalling has an impact on expression of PEA3 Ets transcription factors via the transcriptional repressor capicua. The Biochemical journal 433: 515–525. 10.1042/BJ20101562 21087211 PMC3025492

[30] Bettegowda C, Agrawal N, Jiao Y, Sausen M, Wood LD, et al (2011) Mutations in CIC and FUBP1 contribute to human oligodendroglioma. Science 333: 1453–1455. 10.1126/science.1210557 21817013 PMC3170506

[31] Kawamura-Saito M, Yamazaki Y, Kaneko K, Kawaguchi N, Kanda H, et al (2006) Fusion between CIC and DUX4 up-regulates PEA3 family genes in Ewing-like sarcomas with t(4;19)(q35;q13) translocation. Human molecular genetics 15: 2125–2137. 16717057 10.1093/hmg/ddl136

[32] Cerami E, Gao J, Dogrusoz U, Gross BE, Sumer SO, et al (2012) The cBio cancer genomics portal: an open platform for exploring multidimensional cancer genomics data. Cancer discovery 2: 401–404. 10.1158/2159-8290.CD-12-0095 22588877 PMC3956037

[33] Gao J, Aksoy BA, Dogrusoz U, Dresdner G, Gross B, et al (2013) Integrative analysis of complex cancer genomics and clinical profiles using the cBioPortal. Science signaling 6: pl1 10.1126/scisignal.2004088 23550210 PMC4160307

[34] Sjoblom T, Jones S, Wood LD, Parsons DW, Lin J, et al (2006) The consensus coding sequences of human breast and colorectal cancers. Science 314: 268–274. 16959974 10.1126/science.1133427

[35] Seshagiri S, Stawiski EW, Durinck S, Modrusan Z, Storm EE, et al (2012) Recurrent R-spondin fusions in colon cancer. Nature 488: 660–664. 10.1038/nature11282 22895193 PMC3690621

[36] Lee T, Luo L (2001) Mosaic analysis with a repressible cell marker (MARCM) for Drosophila neural development. Trends in neurosciences 24: 251–254. 11311363 10.1016/s0166-2236(00)01791-4

[37] Queenan AM, Ghabrial A, Schupbach T (1997) Ectopic activation of torpedo/Egfr, a Drosophila receptor tyrosine kinase, dorsalizes both the eggshell and the embryo. Development 124: 3871–3880. 9367443 10.1242/dev.124.19.3871

[38] Karim FD, Rubin GM (1998) Ectopic expression of activated Ras1 induces hyperplastic growth and increased cell death in Drosophila imaginal tissues. Development 125: 1–9. 9389658 10.1242/dev.125.1.1

[39] Ajuria L, Nieva C, Winkler C, Kuo D, Samper N, et al (2011) Capicua DNA-binding sites are general response elements for RTK signaling in Drosophila. Development 138: 915–924. 10.1242/dev.057729 21270056 PMC3035094

[40] Southall TD, Gold KS, Egger B, Davidson CM, Caygill EE, et al (2013) Cell-type-specific profiling of gene expression and chromatin binding without cell isolation: assaying RNA Pol II occupancy in neural stem cells. Developmental cell 26: 101–112. 10.1016/j.devcel.2013.05.020 23792147 PMC3714590

[41] Vogel MJ, Peric-Hupkes D, van Steensel B (2007) Detection of in vivo protein-DNA interactions using DamID in mammalian cells. Nature protocols 2: 1467–1478. 17545983 10.1038/nprot.2007.148 PMC7032955

[42] Kohlmaier A, Fassnacht C, Jin Y, Reuter H, Begum J, et al (2014) Src kinase function controls progenitor cell pools during regeneration and tumor onset in the Drosophila intestine. Oncogene 0. 10.1038/onc.2014.163 24975577

[43] Shwartz A, Yogev S, Schejter ED, Shilo BZ (2013) Sequential activation of ETS proteins provides a sustained transcriptional response to EGFR signaling. Development 140: 2746–2754. 10.1242/dev.093138 23757412

[44] Klambt C (1993) The Drosophila gene pointed encodes two ETS-like proteins which are involved in the development of the midline glial cells. Development 117: 163–176. 8223245 10.1242/dev.117.1.163

[45] O'Neill EM, Rebay I, Tjian R, Rubin GM (1994) The activities of two Ets-related transcription factors required for Drosophila eye development are modulated by the Ras/MAPK pathway. Cell 78: 137–147. 8033205 10.1016/0092-8674(94)90580-0

[46] Brunner D, Ducker K, Oellers N, Hafen E, Scholz H, et al (1994) The ETS domain protein pointed-P2 is a target of MAP kinase in the sevenless signal transduction pathway. Nature 370: 386–389. 8047146 10.1038/370386a0

[47] Morimoto AM, Jordan KC, Tietze K, Britton JS, O'Neill EM, et al (1996) Pointed, an ETS domain transcription factor, negatively regulates the EGF receptor pathway in Drosophila oogenesis. Development 122: 3745–3754. 9012496 10.1242/dev.122.12.3745

[48] Xu C, Kauffmann RC, Zhang J, Kladny S, Carthew RW (2000) Overlapping activators and repressors delimit transcriptional response to receptor tyrosine kinase signals in the Drosophila eye. Cell 103: 87–97. 11051550 10.1016/s0092-8674(00)00107-0

[49] Vivekanand P, Tootle TL, Rebay I (2004) MAE, a dual regulator of the EGFR signaling pathway, is a target of the Ets transcription factors PNT and YAN. Mechanisms of development 121: 1469–1479. 15511639 10.1016/j.mod.2004.07.009

[50] Klaes A, Menne T, Stollewerk A, Scholz H, Klambt C (1994) The Ets transcription factors encoded by the Drosophila gene pointed direct glial cell differentiation in the embryonic CNS. Cell 78: 149–160. 8033206 10.1016/0092-8674(94)90581-9

[51] Roux PP, Blenis J (2004) ERK and p38 MAPK-activated protein kinases: a family of protein kinases with diverse biological functions. Microbiology and molecular biology reviews: MMBR 68: 320–344. 15187187 10.1128/MMBR.68.2.320-344.2004 PMC419926

[52] Carriere A, Cargnello M, Julien LA, Gao H, Bonneil E, et al (2008) Oncogenic MAPK signaling stimulates mTORC1 activity by promoting RSK-mediated raptor phosphorylation. Current biology: CB 18: 1269–1277. 10.1016/j.cub.2008.07.078 18722121

[53] Carriere A, Romeo Y, Acosta-Jaquez HA, Moreau J, Bonneil E, et al (2011) ERK1/2 phosphorylate Raptor to promote Ras-dependent activation of mTOR complex 1 (mTORC1). The Journal of biological chemistry 286: 567–577. 10.1074/jbc.M110.159046 21071439 PMC3013016

[54] Ma L, Chen Z, Erdjument-Bromage H, Tempst P, Pandolfi PP (2005) Phosphorylation and functional inactivation of TSC2 by Erk implications for tuberous sclerosis and cancer pathogenesis. Cell 121: 179–193. 15851026 10.1016/j.cell.2005.02.031

[55] Tomlins SA, Laxman B, Dhanasekaran SM, Helgeson BE, Cao X, et al (2007) Distinct classes of chromosomal rearrangements create oncogenic ETS gene fusions in prostate cancer. Nature 448: 595–599. 17671502 10.1038/nature06024

[56] Chi P, Chen Y, Zhang L, Guo X, Wongvipat J, et al (2010) ETV1 is a lineage survival factor that cooperates with KIT in gastrointestinal stromal tumours. Nature 467: 849–853. 10.1038/nature09409 20927104 PMC2955195

[57] Jane-Valbuena J, Widlund HR, Perner S, Johnson LA, Dibner AC, et al (2010) An oncogenic role for ETV1 in melanoma. Cancer research 70: 2075–2084. 10.1158/0008-5472.CAN-09-3092 20160028 PMC2846410

[58] Pratilas CA, Taylor BS, Ye Q, Viale A, Sander C, et al (2009) (V600E)BRAF is associated with disabled feedback inhibition of RAF-MEK signaling and elevated transcriptional output of the pathway. Proceedings of the National Academy of Sciences of the United States of America 106: 4519–4524. 10.1073/pnas.0900780106 19251651 PMC2649208

[59] Hollenhorst PC, Ferris MW, Hull MA, Chae H, Kim S, et al (2011) Oncogenic ETS proteins mimic activated RAS/MAPK signaling in prostate cells. Genes & development 25: 2147–2157. 22012618 10.1101/gad.17546311 PMC3205585

[60] Micchelli CA, Perrimon N (2006) Evidence that stem cells reside in the adult Drosophila midgut epithelium. Nature 439: 475–479. 16340959 10.1038/nature04371

[61] Romani P, Bernardi F, Hackney J, Dobens L, Gargiulo G, et al (2009) Cell survival and polarity of Drosophila follicle cells require the activity of ecdysone receptor B1 isoform. Genetics 181: 165–175. 10.1534/genetics.108.096008 19015542 PMC2621165

[62] Bischof J, Bjorklund M, Furger E, Schertel C, Taipale J, et al (2013) A versatile platform for creating a comprehensive UAS-ORFeome library in Drosophila. Development 140: 2434–2442. 10.1242/dev.088757 23637332

[63] Brand AH, Perrimon N (1993) Targeted gene expression as a means of altering cell fates and generating dominant phenotypes. Development 118: 401–415. 8223268 10.1242/dev.118.2.401

[64] Dutta D, Xiang J, Edgar BA (2013) RNA expression profiling from FACS-isolated cells of the Drosophila intestine. Current protocols in stem cell biology 27: Unit 2F 2 10.1002/9780470151808.sc02f02s27 24510286

[65] Shaw RL, Kohlmaier A, Polesello C, Veelken C, Edgar BA, et al (2010) The Hippo pathway regulates intestinal stem cell proliferation during Drosophila adult midgut regeneration. Development 137: 4147–4158. 10.1242/dev.052506 21068063 PMC2990206

[66] Anders S, Pyl PT, Huber W (2015) HTSeq-a Python framework to work with high-throughput sequencing data. Bioinformatics 31: 166–169. 10.1093/bioinformatics/btu638 25260700 PMC4287950

[67] Robinson MD, McCarthy DJ, Smyth GK (2010) edgeR: a Bioconductor package for differential expression analysis of digital gene expression data. Bioinformatics 26: 139–140. 10.1093/bioinformatics/btp616 19910308 PMC2796818

[68] Benjamini Y, and Hochberg Y (1995) Controlling the False Discovery Rate—a Practical and Powerful Approach to Multiple Testing. Journal of the Royal Statistical Society Series B-Methodological 57: 289–300.

[69] Papagiannouli F, Schardt L, Grajcarek J, Ha N, Lohmann I (2014) The Hox gene Abd-B controls stem cell niche function in the Drosophila testis. Developmental cell 28: 189–202. 10.1016/j.devcel.2013.12.016 24480643

[70] Langmead B, Salzberg SL (2012) Fast gapped-read alignment with Bowtie 2. Nature methods 9: 357–359. 10.1038/nmeth.1923 22388286 PMC3322381

[71] Southall TD, Davidson CM, Miller C, Carr A, Brand AH (2014) Dedifferentiation of neurons precedes tumor formation in Lola mutants. Developmental cell 28: 685–696. 10.1016/j.devcel.2014.01.030 24631403 PMC3978655

