# Supplementary figures and images for: EGFR/Ras Signaling Controls Drosophila Intestinal Stem Cell Proliferation via Capicua-Regulated Genes
Source: PLoS Genet. 2015 Dec 18;11(12):e1005634. doi: 10.1371/journal.pgen.1005634 (PMC4684324; doi:10.1371/journal.pgen.1005634)

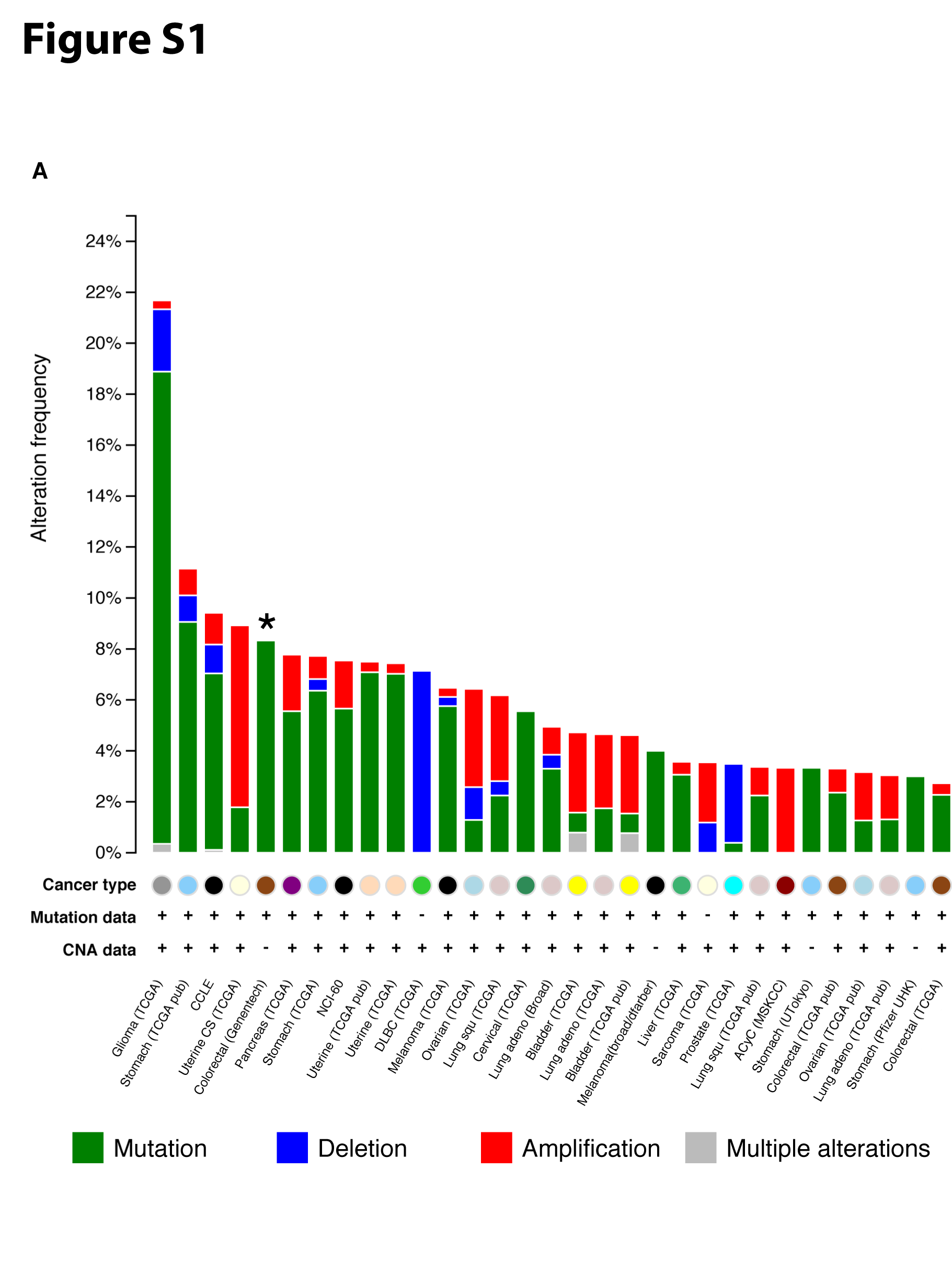

Supplement: S1 Fig — The figure was reproduced from the cBioPortal for Cancer Genomics web page and modified to show only cancers with >3.3% alteration frequency. Asterisks mark colorectal cancer data from Genentech [35]. (TIF) [file pgen.1005634.s001.tif]

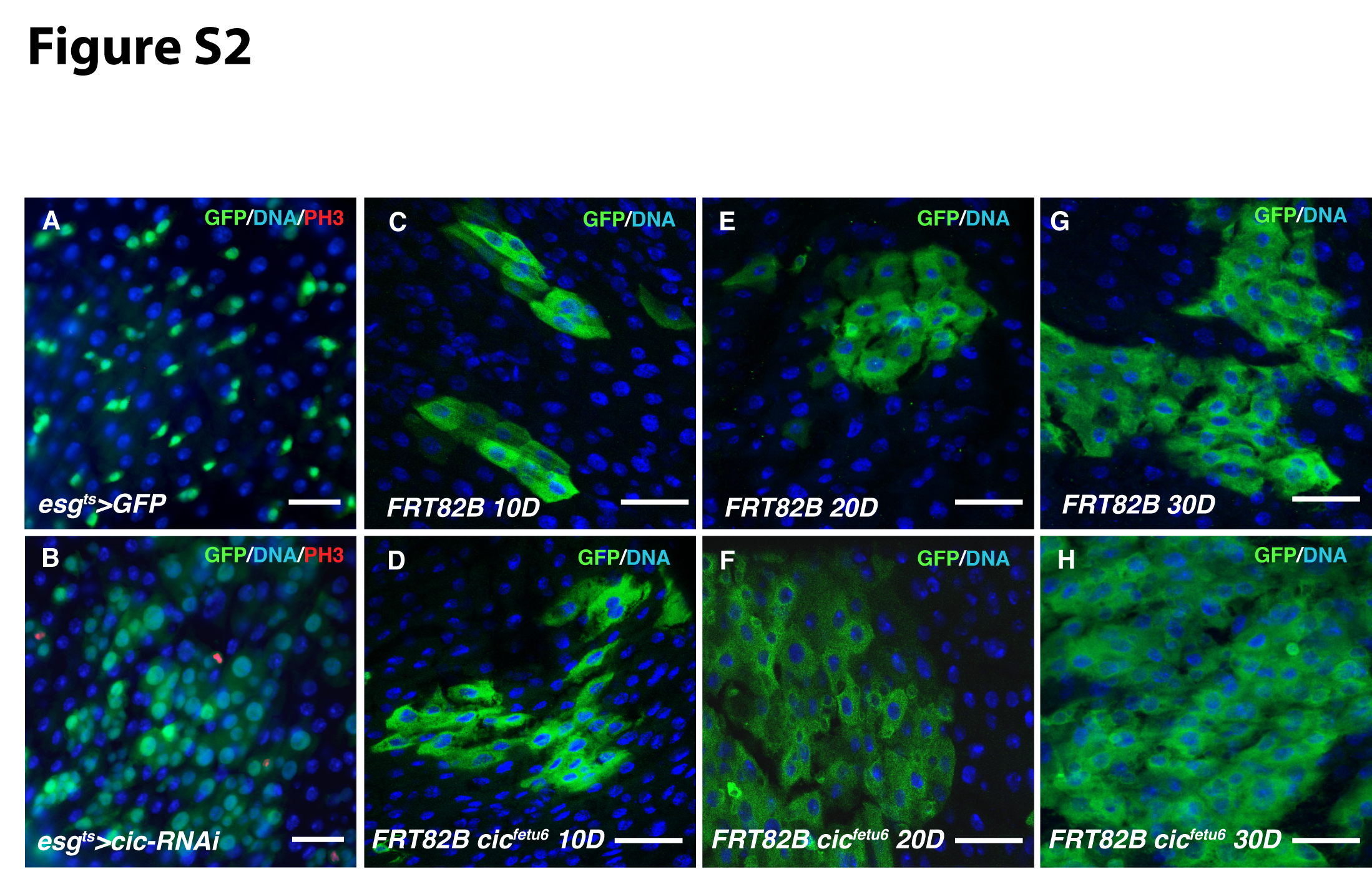

Supplement: S2 Fig — (A, B) RNAi-mediated depletion of Cic in ISCs and EBs using the esg ts system. esg+ progenitor cells (green), PH3+ (red) nuclear DNA (blue). (A) Control adult midgut (B) Cic knock down midgut after 4 days induction 29°C. Scale bars represent 50μm. (C-H) Cic mutant clones were analyzed using the MARCM system. ISC clones (green), DNA stained with DAPI (blue). Control (C, E, G) and mutant (D, F, H) ISC clones were induced with the MARCM system and examined 10 days, 20 days and 30 days later. Mutant ISCs divided faster and generated bigger clones. Scale bars represent 100μm. (TIF) [file pgen.1005634.s002.tif]

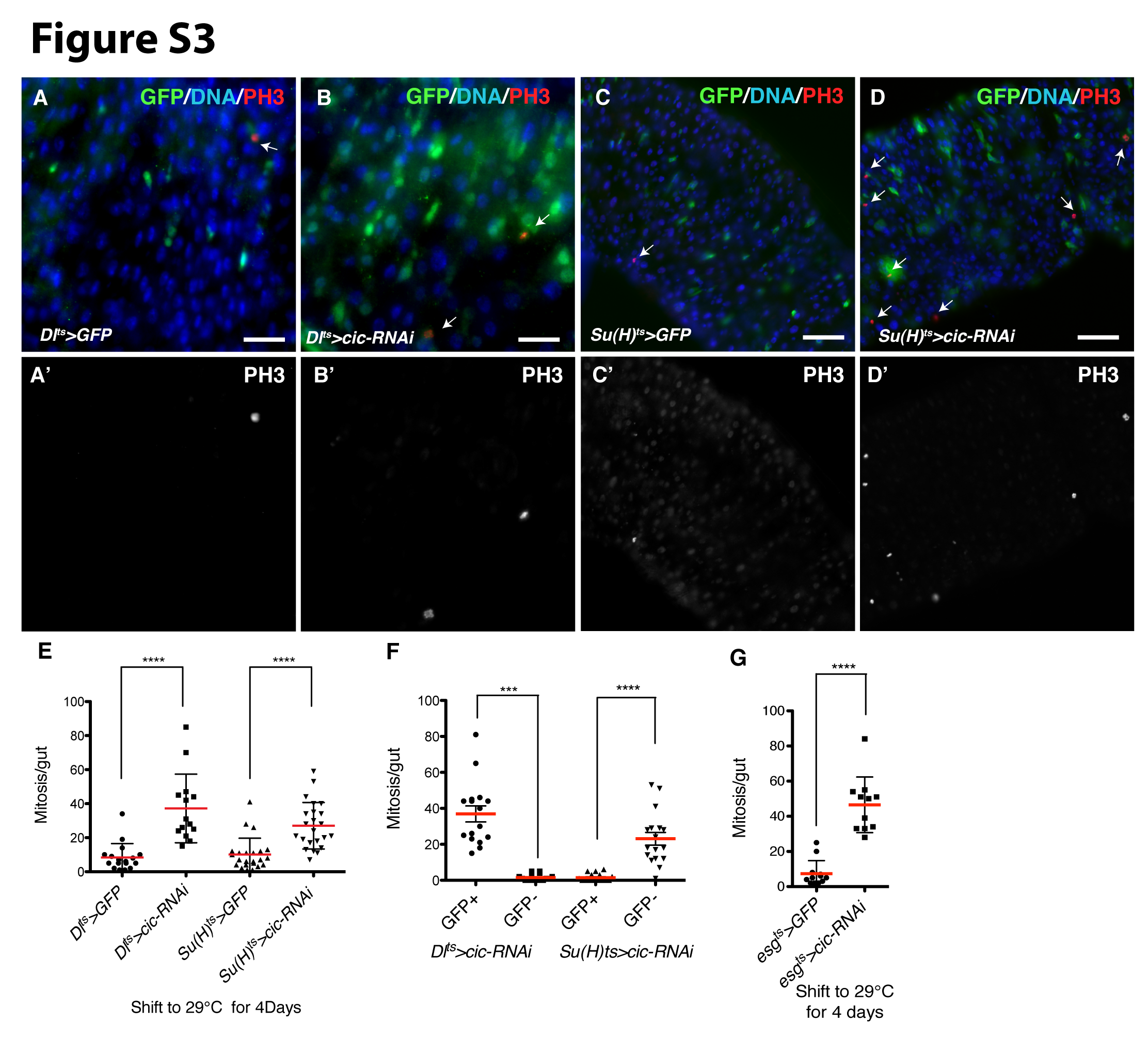

Supplement: S3 Fig — (A, B) RNAi-mediated depletion of Cic in ISCs using the Dl ts system. ISCs are marked by GFP (green). Sample was also stained with anti-PH3 to detect mitoeses (red) and DAPI to detect nuclear DNA (blue). (A) Control adult midgut, (B) Cic depleted midgut after 4 days induction at 29°C. Dramatic increases in the number of GFP positive cells were observed in cic depleted midguts, as was large increase in ISC mitoses. (C, D) RNAi-mediated depletion of Cic in EBs using the Su(H) ts system. EB cells are marked by GFP (green). Samples were also stained with anti-PH3 (red) and DAPI (blue). (C) Control adult midgut (D) Cic depleted midgut after 4 days induction 29°C. Increases in the number of GFP positive cells and mitoses were observed in cic knockdown midguts. (E) Midguts as in A-C were scored for PH3+ cells after 4 days of induction of cic-RNAi in ISCs or EBs. (F) After 4 days induction of cic-RNAi in ISCs or EBs, midguts were scored for GFP+ or GFP- mitotic cells. Most mitotic cells were GFP+ when cic-RNAi was induced in ISCs using the Dl ts system, whereas in midguts in which cic was depleted in EBs, most of the mitotic cells were GFP- and likely ISCs. This indicates a non-cell autonomous effect. (G) Midguts were scored for PH3+ cells after 4 days of induction of cic-RNAi using the esg ts system, which targets gene expression to ISCs and EBs. Dramatic increases in the number of GFP positive cells were observed in cic knockdown midguts as was a large increase in ISC mitoses. Statistical significance was determined by Student’s t test (*p<0.05, **p<0.01, ***p<0.001, ****p<0.0001). Error bars in each graph represent standard deviation. Scale bars represent 20μm. (TIF) [file pgen.1005634.s003.tif]

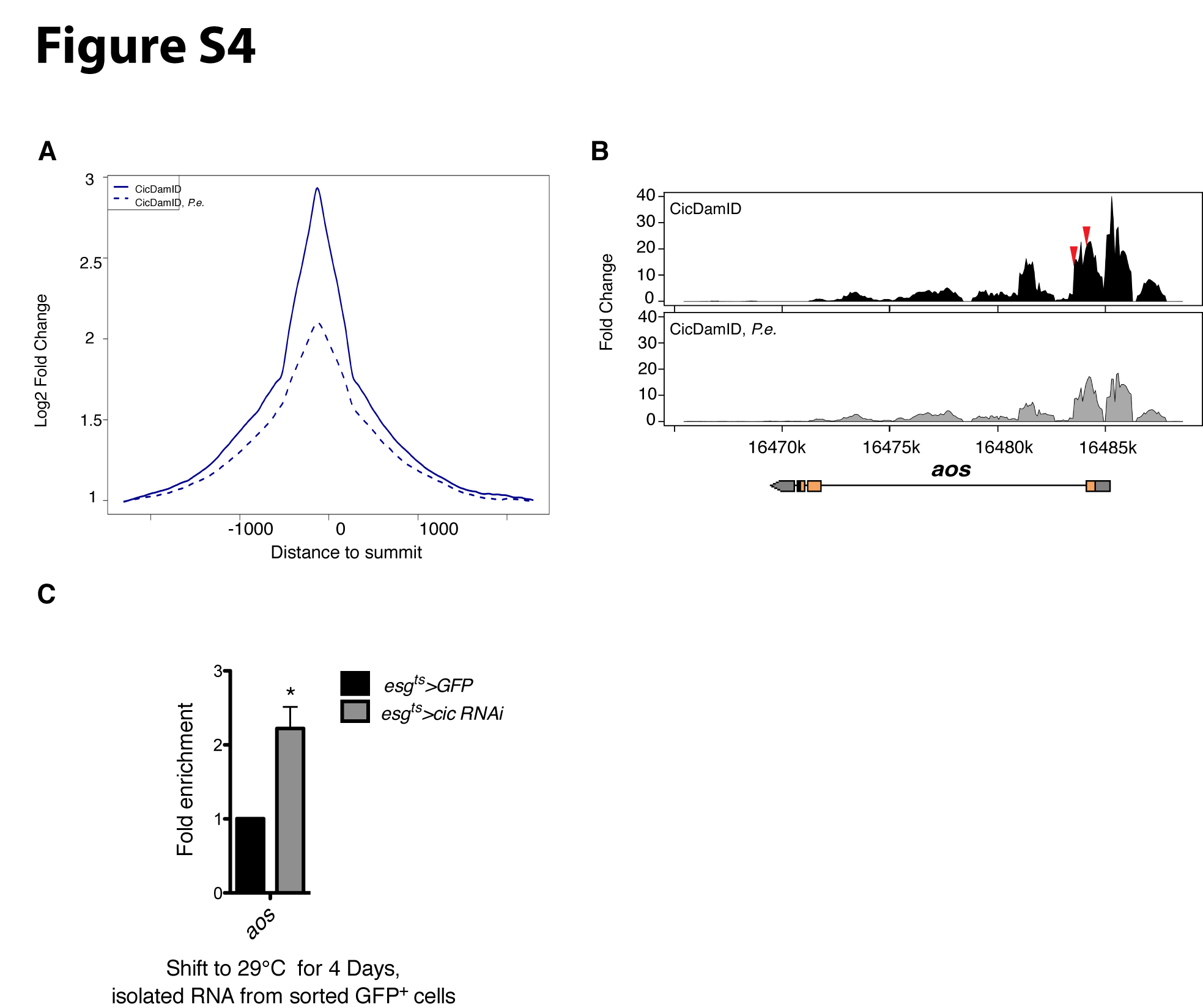

Supplement: S4 Fig — (A) Graph showing fold change of peaks from Cic-DamID and P.e. infected Cic-DamID samples. (B) Cic binding sites in the aos locus from Cic-DamID-Seq using midgut ISCs. The black peaks are from control animals, and the grey peaks are from P.e. infected animals. Plot represents the log2 ratio between the Dam-fusion signal and the Dam-only signal. Red arrows point out TGAATG(G/A)A motifs. The aos transcription unit is shown below the graph. Yellow boxed regions indicate the ORF. (C) mRNA expression ratio change of aos was analyzed by qRT-PCR and normalized to β-Tub and Rp49 with non-amplified mRNA from FACS-sorted progenitor cells. (TIF) [file pgen.1005634.s004.tif]

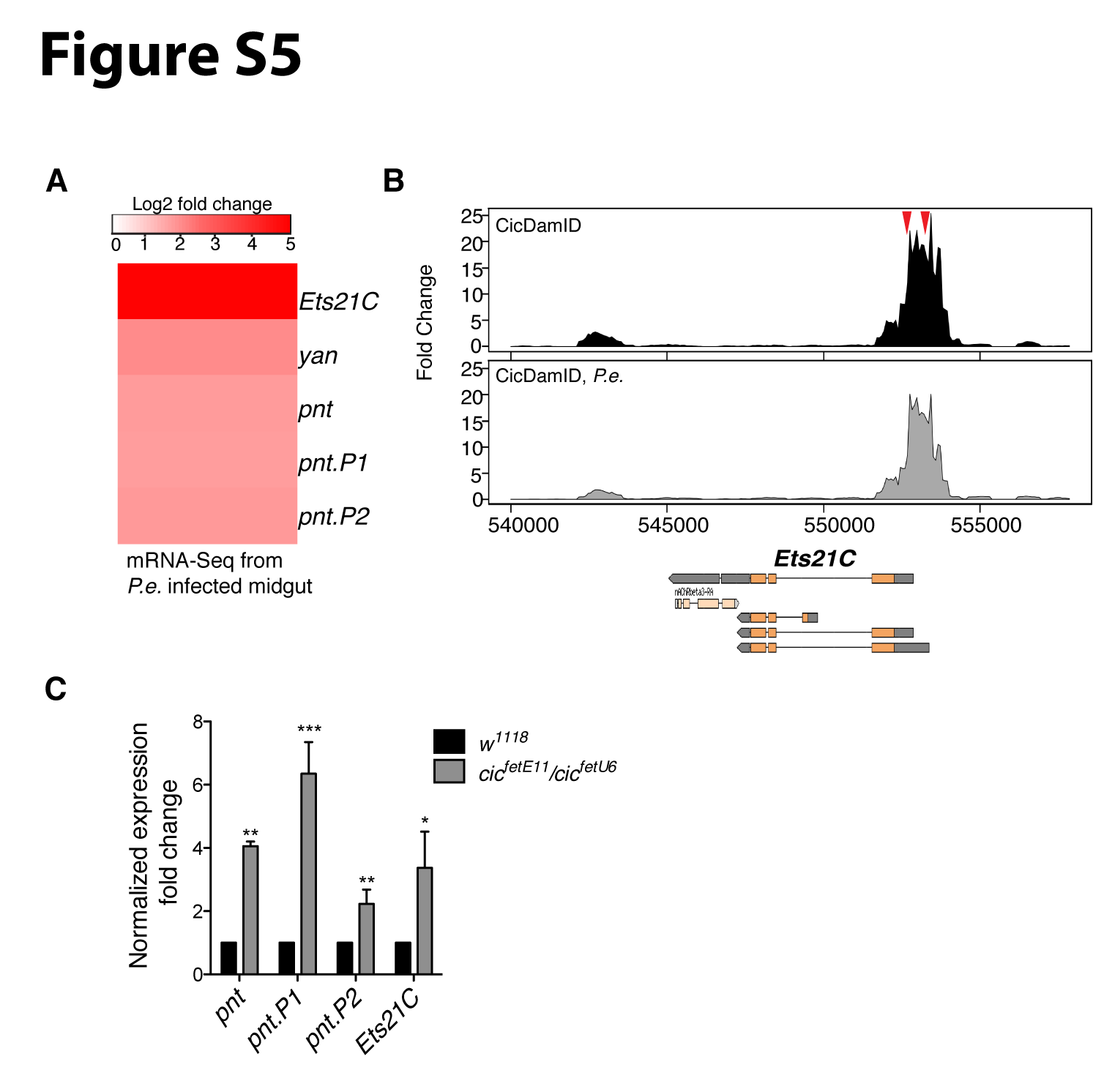

Supplement: S5 Fig — (A) mRNA expression heatmap of Ets transcription factors, showing fold change inductions from RNA-Seq data from whole midguts upon 6 hours P.e. infection. (B) Cic binding sites in the Ets21C locus from Cic-DamID-Seq from esg+ cells. Black peaks are from control samples and grey peaks are from P.e. infected midguts. The Y-axis represents the log2 ratio of the Cic-Dam fusion signal to the Dam-only signal. Red arrows point out TGAATG(G/A)A motifs. (C) Normalized mRNA expression fold change of pnt, pntP1, pntP2 and Ets21C in cic transheterozygous mutant midguts. (TIF) [file pgen.1005634.s005.tif]

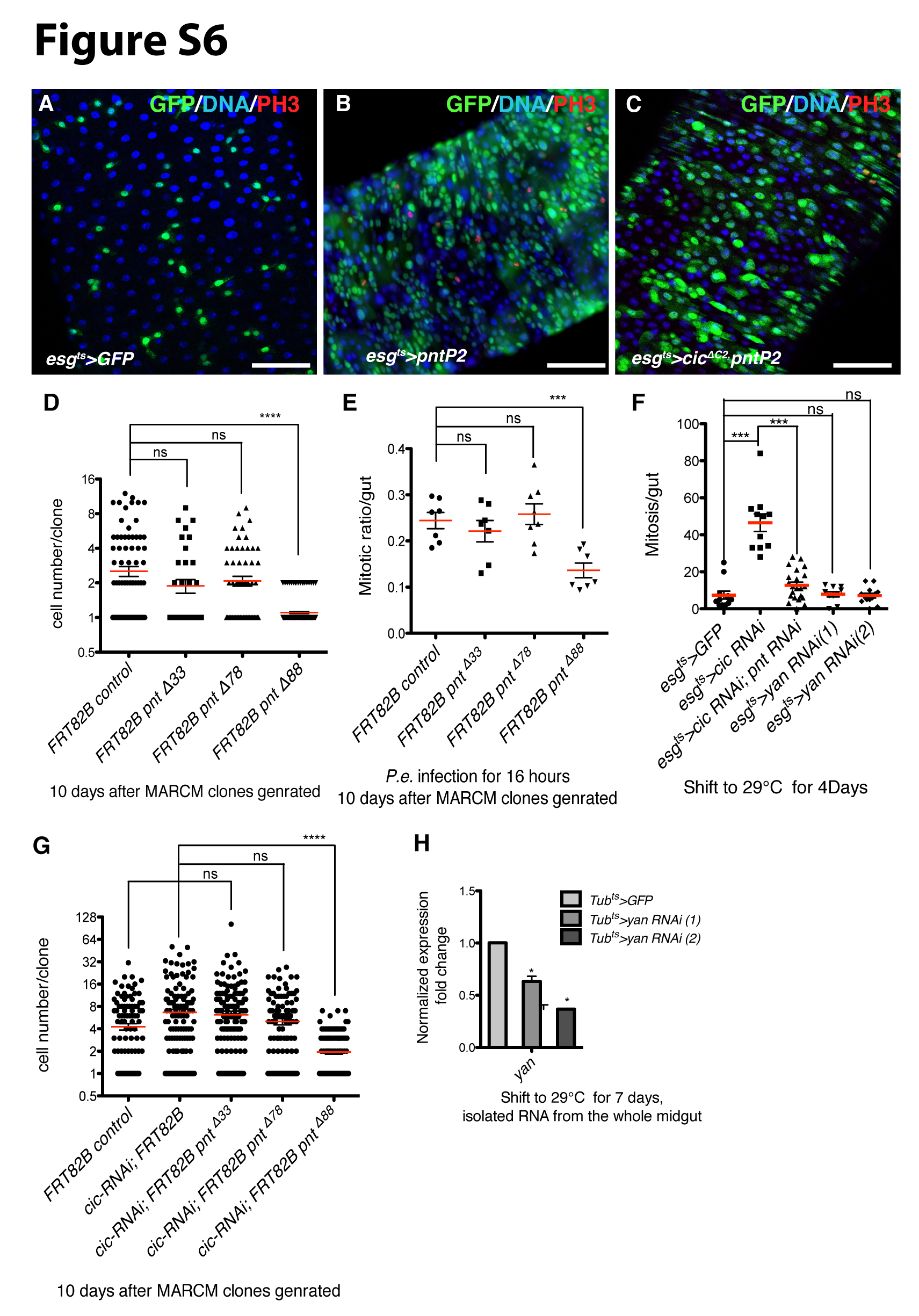

Supplement: S6 Fig — (A–C) Effect of pntP2 overexpression on ISC proliferation. Transgene expression was induced using the esg ts system at 29°C for 4 days. Samples were stained with anti-GFP (green), anti-PH3(red) and DAPI (blue) to mark DNA. (A) Control adult midgut. (B) pntP2 overexpressing midgut. The pntP2 over expressing midgut had more GFP+ ISCs and EBs (green). (C) pntP2 and cic ΔC2 over expressing midgut. GFP positive progenitor cells were still able to proliferate in the pnt, cic ΔC2 over-expressing midgut. (D) pnt mutant clones analyzed by the MARCM system. The size of the clones was quantified by counting cell numbers per clone. pnt Δ33 is a pntP1 specific mutant allele, pnt Δ78 is pntP2 specific mutant allele and pnt Δ88 is pnt null mutant allele that affect both isoforms. Only the pnt Δ88 detectably suppressed clone expansion. (E) Mitotic ratio of the pnt mutant clones was scored by calculating the average number of mitoses in each clone. (F) Quantification of ISC mitoses (PH3 positive cells) in pnt and cic depleted midguts or yan depleted midguts, using esg ts system. Fewer mitotic ISCs were observed in the pnt and cic double knock down midgut than in the cic knockdown midguts, showing that pnt is required downstream of cic. Yan depletion had no effect on ISC proliferation. (G) pnt mutant clones were generated in a cic depleted background using the MARCM system. The size of the clones was quantified by counting cell numbers per clone. Only the pnt Δ88 null allele suppressed the growth of cic-depleted ISC cell clones. (H) yan expression ratio as measured by qRT-PCR in yan-depleted midguts, using two different yan-RNAi lines. Statistical significance was determined by Student’s t test (*p<0.05, **p<0.01, ***p<0.001, ****p<0.0001). Error bars in each graph represent standard deviation. Scale bars represent 50μm. (TIF) [file pgen.1005634.s006.tif]

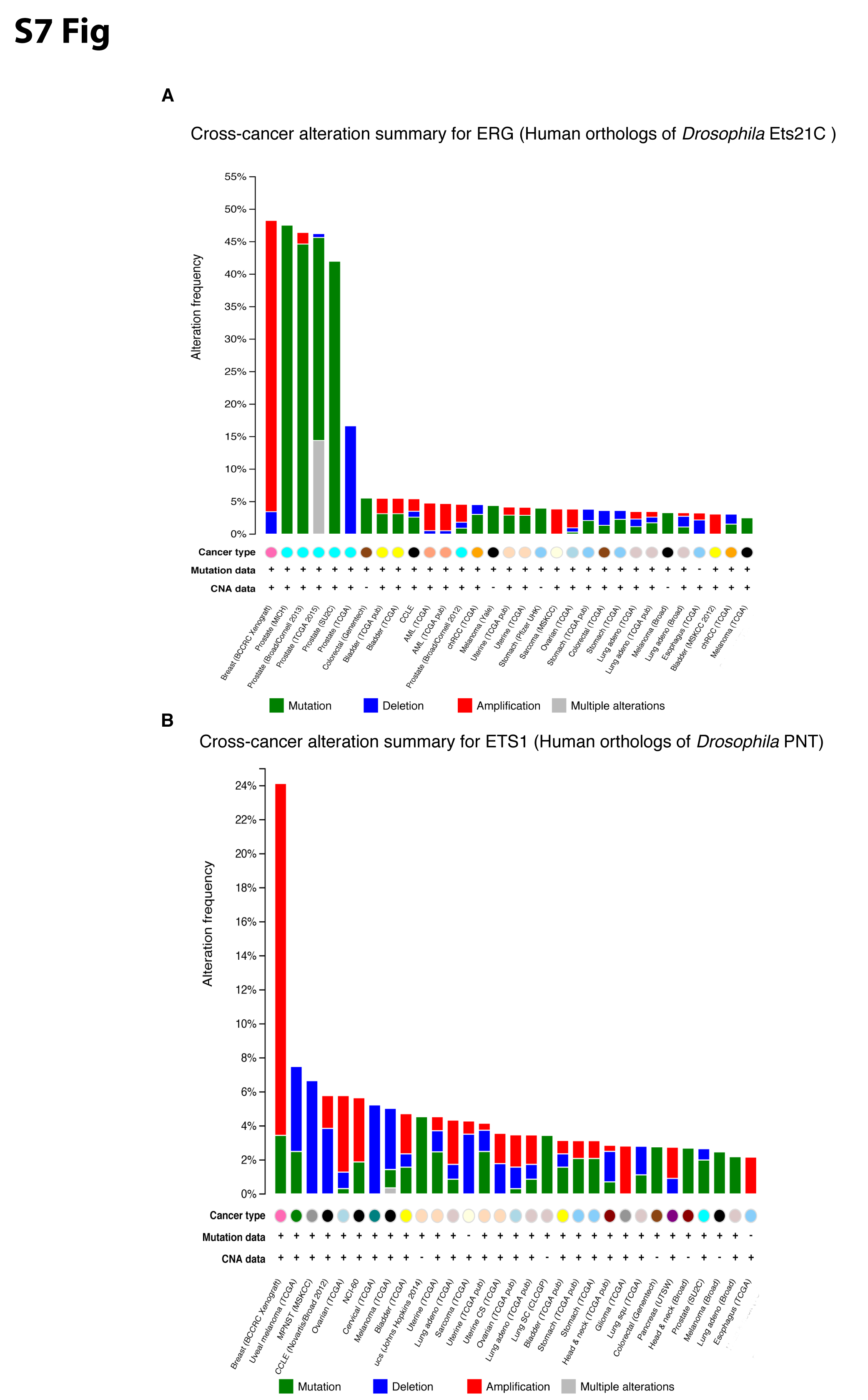

Supplement: S7 Fig — The figure was reproduced from the cBioPortal for Cancer Genomics web page and modified to show only cancers with >3.3% alteration frequency. (A) Cross-cancer alteration summary for EGR (the human orthologs of Drosophila Ets21C). (B) Cross-cancer alteration summary for EGR (the human orthologs of Drosophila Pnt). (TIF) [file pgen.1005634.s007.tif]
