# Supplementary material for: EGFR/Ras Signaling Controls Drosophila Intestinal Stem Cell Proliferation via Capicua-Regulated Genes
Source: PLoS Genet. 2015 Dec 18;11(12):e1005634. doi: 10.1371/journal.pgen.1005634 (PMC4684324; doi:10.1371/journal.pgen.1005634)
Supplement: S4 Table — Information of well-known growth promoters was listed in the table with their log2 Fold change in RNA-Seq and significance (p-value with Benjamini-Hochberg correction) and numbers of Cic Dam-ID peaks, found in their introns or within 5kb range of the transcription start site. Genes that have Cic binding sites are shaded grey. (PDF) [file pgen.1005634.s011.pdf]

S4 Table

S4 Table. Growth regulatros in RNA-seq and DamID-seq					
Gene Name	FlyBase ID	log2 Fold change in RNA-Seq	p-value	FDR	Number of peaks from DamID-Seq
Egfr	FBgn0003731	0.396823326	0.003130705	0.026285491	5
Pvr	FBgn0032006	0.08367124	0.550648602	0.760870425	0
bnl	FBgn0014135	0.341343527	0.01597985	0.086804798	1
InR	FBgn0013984	0.8173407	1.07E-07	6.73E-06	1
Akt1	FBgn0010379	0.491905074	6.21E-05	0.001313506	1
Pi3K21B	FBgn0020622	0.076680225	0.521164182	0.738806317	1
Pi3K59F	FBgn0015277	-0.564612619	7.53E-06	0.000245288	0
Pi3K68D	FBgn0015278	-0.225577118	0.092345618	0.278946255	0
Pi3K92E	FBgn0015279	0.163285775	0.289577478	0.544273858	0
Rheb	FBgn0041191	-0.325749099	0.013170328	0.074677832	1
Pten	FBgn0026379	-0.063052057	0.7286371	0.869404353	0
S6k	FBgn0015806	-0.256735962	0.082909218	0.259920237	0
dm(Myc)	FBgn0262656	0.198450761	0.162583155	0.389608008	1
Tif-IA	FBgn0032988	0.199704149	0.117858829	0.321230939	0
Brf	FBgn0038499	0.181791338	0.47121442	0.703756109	0
Maf1	FBgn0058196	0.039614682	0.775112954	0.893548434	0
Src64B	FBgn0262733	-0.159194964	0.200881154	0.440306122	0
Src42A	FBgn0264959	-0.015371949	0.912296323	0.963752427	1
Ack	FBgn0028484	0.342997507	0.017618912	0.092770616	1
Btk29A	FBgn0003502	0.084274149	0.807789359	0.909543003	1
CycD	FBgn0010315	0.147362775	0.598057015	0.791401204	0
Cdk4	FBgn0004107	0.429571817	0.018271328	0.09483809	2
crb	FBgn0259685	-0.068538173	0.655680161	0.830976355	1
yki	FBgn0034970	-0.185011084	0.138529244	0.354259222	1
rDNA loci	—	not tested	not tested	not tested	0
